# Using electricity tariffs and thermal comfort management to promote residential energy decarbonization

**DOI:** 10.1016/j.isci.2025.112631

**Published:** 2025-05-12

**Authors:** Andrea Vecchi, Michael John Brear

**Affiliations:** 1 Department of Mechanical Engineering, The University of Melbourne, Parkville, VIC 3010, Australia; 2 Melbourne Energy Institute (MEI) and Department of Mechanical Engineering, The University of Melbourne, Parkville, VIC 3010, Australia

**Keywords:** Energy policy

## Abstract

Household thermal demand supply through electricity could reduce emissions, but also compromise the operation of electricity distribution networks. In this work, suitable electricity tariff designs and household thermal management are investigated, individually and in combination, as ways to enhance network operability, support electrification, and improve system-level performance. Optimization results from eight combinations of building type, vintage, and climates in Australia show considerable social benefits resulting from the measures explored. Electricity tariffs that include a demand charge contain peak loads without increasing household costs. Furthermore, emission and cost savings from looser thermostat setpoints and building pre-heating/cooling are demonstrated. These are also found to reduce the upfront investment in household electrification to levels comparable with current, dual-fuel (electricity and natural gas) practices. Subject to the degree of occupants’ acceptance and policy adoption, a significant potential to mitigate, and even avoid, anticipated network strain, while tackling pressing affordability and decarbonization challenges is demonstrated.

## Highlights

• Optimal household response under comfort constraints across buildings and climates
• Assessment of electricity tariffs and comfort management to support electrification
• System-level performance is improved without increasing household costs
• Potential to mitigate and avoid network strain and affordability issues is revealed

## Graphical abstract

**Figure:**
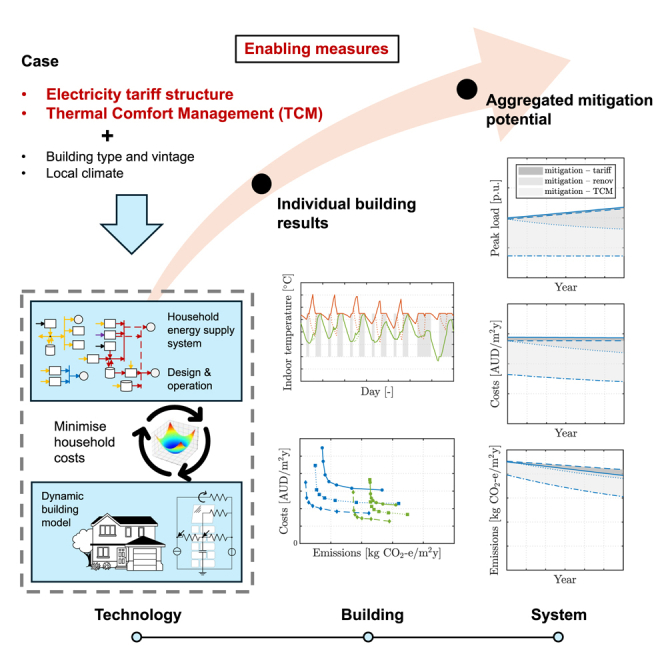

## Introduction

Energy supply in buildings is currently responsible for about 18% of greenhouse gas (GHG) emissions worldwide.^1^ The electrification of household equipment can enable the supply of low-carbon energy to this sector,^2^ thus contributing to its decarbonization. Electric appliances typically have high efficiency and flexibility^3^ and can benefit from the growing decarbonization of most electricity networks, along with the uptake of distributed energy resources (DER) that generate and store energy onsite.^4^ As a result, and often aided by policies aimed at reducing the impact of upfront costs on consumers,^5^ household electrification is an ongoing trend in several economies.^6^

Although electricity supplying larger shares of buildings’ thermal demand can be environmentally preferable^7^ and yield economic benefits by reducing both customer^8^^,^^9^ and system^10^^,^^11^ costs, challenges can still arise from the resulting higher utilization of electrical infrastructure. Some studies project aggregated peak loads to even double by 2050^12^ and potentially earlier^13^ from 2020 levels, imposing greater requirements on electrical distribution networks,^14^ and calling for well-orchestrated building electrification to alleviate these issues.^15^ Electrification of energy supply in buildings also usually involves significant investments in renewables to generate the required, clean electricity, whether through DER or at utility scale.^16^

Demand response is one option for mitigating such challenges, and involves shifting and shedding building load, potentially through smart thermal comfort management (TCM),^17^ to benefit either the system or the occupant, and preferably both.^18^ Particularly when coupled with enhanced building efficiency, demand-side measures can be effective in limiting burdens on the power system.^19^ For example, building pre-cooling, lighting, and window control have been shown to yield more than a 40% reduction in the peak cooling load of two offices in a temperate climate.^20^ In winter, heat pumps can also be flexibly operated to stabilize the network voltage.^21^ These and similar strategies exploit the building’s thermal mass as a source of flexibility^22^ and all entail that acceptable indoor comfort can be maintained. Thus, the impact of each strategy will differ across climates and buildings, and its assessment should consider representative building practices. Even so, additional measures may still be needed to limit future electrical network augmentation and associated costs.^23^

Suitable electricity tariffs may be one. By reflecting network costs, tariffs can encourage electricity use at times of greater generation and/or availability of network capacity, thus reducing peak loads and valuing flexibility.^24^^,^^25^ Additionally, electricity tariffs affect demand peaks by influencing technology selection and sizing.^26^^,^^27^ Any assessment of tariffs’ potential to support electrification should therefore account for local climate conditions and also differentiate whether tariffs are set after or can indeed influence the design of the building energy system. On the other hand, studies^28^^,^^29^ demonstrate a rebound in electricity demand and consumption outside designated peak windows as a potential shortcoming, which can result in unintended system expansion.^30^ Hence, also electricity tariff design affects building decarbonization by either relieving or worsening associated system-level impacts.

Overall, published studies typically consider single approaches to electrification, and therefore do not normally capture how.

(1) different measures may be more or less suitable in different climates and for different building types;
(2) the interdependency of different measures may result in different economic, technical, and environmental benefits, and thus different potentials to support electrification when considered *together*; and
(3) the highlighted drawbacks may be reduced by the integration of these different measures.

Furthermore, potential *system benefits* such as aggregated emissions and peak load reductions should be a consequence of rational decisions made *by the homeowner or occupant*; that is, by minimizing *their* total costs. This, for instance, leads to different flexibility or DER options becoming prospective at different peak-to-off peak price ratios^31^ and may not always align with system operability goals.^32^ The design of electricity tariffs is therefore key to resolving this tension between system-wide and individual benefits. Finally, the flexibility and load mitigation potential of different strategies when considering building operations only, as opposed to design and operations together, remains largely unexplored.

Therefore, this work addresses the research question: “*How might electricity tariff design and household demand response be used, individually or in combination, to improve system-level performance and* support *building electrification?.”* An integrated platform is used to simulate the dynamic energy demand of eight building archetypes (Table 1) – representative of old and new buildings in a temperate (Melbourne) and a sub-tropical (Brisbane) climate in Australia – and optimize the design and operation of the technology mix that supplies them. Electricity tariff structures combining different time-based pricing and a demand charge levied on the peak load requested by the household, as well as TCM strategies that allow progressively looser thermostat setpoints and building pre-heating/cooling, are tested for their effect on the optimal results. With this framework, the role of electricity tariff structure, TCM and building energy efficiency on the potential reductions in costs, emissions, and peak loads is studied. Finally, results are projected using plausible electrification trajectories and building renovation rates to illustrate the impact that these measures can have on mitigating future peak loads on residential feeders, thus alleviating the requirements imposed on the electricity network.

**Table 1:** Key features, thermophysical properties, and representative dynamic building model parameters of the representative households considered in this work

Building characteristics	Melbourne – Victoria				Brisbane – Queensland			
Type	House	House	Apartment	Apartment	House	House	Apartment	Apartment
Vintage	Old	New	Old	New	Old	New	Old	New
Floor surface [m^2^]	124	157	124	67	98	125	86	91
Height [m]	2.4	2.4	2.4	2.4	2.4	2.4	2.4	2.4
Façade [m^2^]	75	81	84	54	70	78	69	62
Windows/floor [%]	26	25	18	37	25^a^	23	23^a^	33
Windows surface [m^2^]	32	39	23	25	25	29	20	30
Construction	Heavy	Medium	Heavy	Medium	Light	Very light	Light	Very light
Wall type	Brick veneer	Brick veneer	Cavity masonry	Lightweight cladding^a^	Brick veneer^a^	Brick veneer	Cavity masonry^a^	Hollow concrete
Wall R [m^2^K/W]	2.2	4.8	2.1	4.8	1.2	3.0	2.0	2.9
Wall convective/radiative resistance [m^2^K/W]	0.13	0.13	0.13	0.13	0.13	0.13	0.13	0.13
Roof/ceiling type	Tiled	Metal	Concrete plenum^a^	Concrete	Tiled^a^	Metal	Concrete plenum^a^	Concrete
Roof/ceiling R [m^2^K/W]	1.0	6.2	1.0	7.5	1.5	5.9	1.0	5.1
Ceiling convective/radiative resistance [m^2^K/W]	0.1	0.1	0.1	0.1	0.1	0.1	0.1	0.1
Floor type	Timber	Waffle pod	Timber^a^	Concrete	Timber^a^	Waffle pod	Waffle pod^a^	Concrete
Floor R [m^2^K/W]	1.2	3.9	2.5	5.4	0.7	2.4	1.5	4.4
Floor convective/radiative resistance [m^2^K/W]	0.17	0.17	0.17	0.17	0.17	0.17	0.17	0.17
Windows type	Timber single	Timber double	PVC single	PVC double	Timber single	Timber double	PVC single	PVC double
Windows U [W/m^2^K]	5.4	2.9	5.4	2.9	5.4	2.9	5.4	2.9
Windows SHGC [-]	0.49	0.42	0.49	0.42	0.49	0.42	0.49	0.42
Ventilation rate [1/m^3^h]	0.95	0.35	0.95	0.35	0.95	0.35	0.95	0.35
2022 NatHERS Star band	2	6.5	2.5	6.5	4.5	6.5	5	6.5

**Representative dynamic building model parameters**								

$R^{th,1}$ [K/kW]	0.4	0.3	0.4	0.7	0.5	0.4	0.5	0.5
$R^{th,2}$ [K/kW]	3.8	12.1	4.7	30.7	3.6	10.1	5.6	16.6
$R^{th,3}$ [K/kW]	5.8	8.9	8.1	13.8	7.5	11.9	9.2	11.6
$R^{th,4}$ [K/kW]	10.6	22.8	10.6	53.3	13.4	28.6	15.3	39.3
$C^{th}$ [kJ/K]	32162	25872	32292	11063	10772	9982	9482	7265

a based on national average by building type and vintage, for lack of reported data with state granularity.

## Results and discussion

### Indoor temperature for various buildings, electricity tariffs, and thermal comfort management strategies

**Figure 1:**
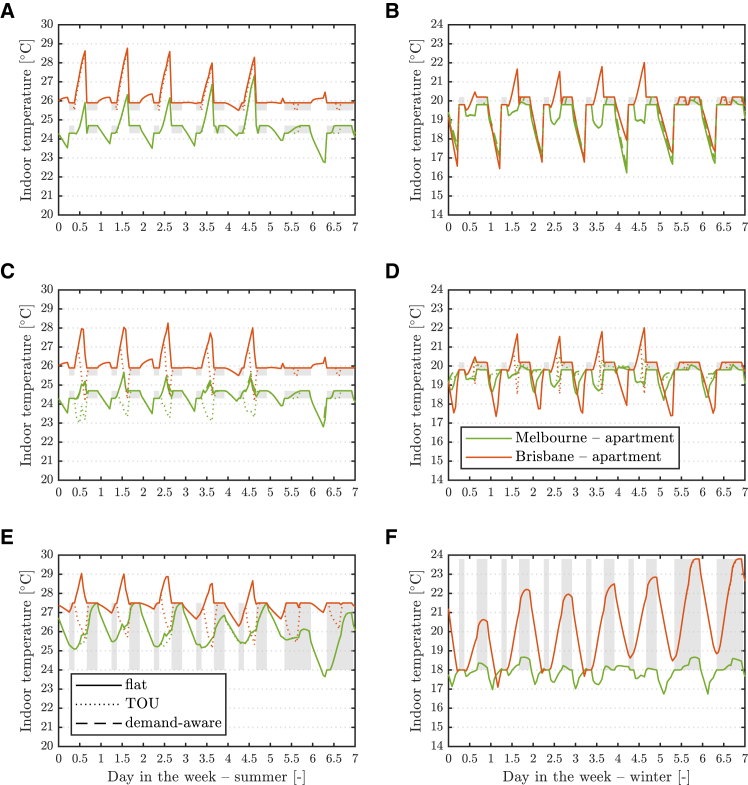
Indoor temperatures without DER for various electricity tariffs and TCM strategies Building indoor temperature evolution for one week in summer (left) and winter (right) at two sites without DER and for three electricity tariffs with tight setpoint and no pre-heating/cooling (top), tight setpoint with pre-heating/cooling (middle), and loose setpoint with pre-heating/cooling (bottom). Only key results for new apartments are shown. The shaded regions identify the thermostat setpoint range and timing. Note the different scales for the y axis.

We first consider the evolution of indoor temperature with our dynamic building model applied to the eight building types without DER. Figure 1 demonstrates that local climate and electricity tariffs both affect the homeowner’s optimal demand response. Under a time-of-use (TOU) tariff, building pre-cooling is used in summer when it is allowed, in the central part of the day, to shift electricity consumption away from higher-price windows. A looser thermostat setpoint (Figure 1E) reduces the required pre-cooling in Melbourne but prompts little change in Brisbane, where larger solar gains still justify pre-cooling. In winter, morning pre-heating occurs in Melbourne for all tariffs. This allows for downsized thermal supply equipment, particularly in older buildings, with about a 50% smaller natural gas boiler capacity projected for detached houses. The building's indoor temperature evolution in these cases is shown in Figure S1. Households in Brisbane with a TOU tariff still operate pre-cooling in the afternoon on most winter days, although a looser thermostat setpoint (Figure 1F) would result in morning heating and little, if any, cooling.

Neither flat nor demand-aware (i.e., including a demand charge) tariffs are found to promote demand response via pre-heating/cooling. Indeed, energy supply outside setpoint windows results in extra thermal losses and if the energy price is constant for the former tariff, it brings no rewards to the homeowner. Conversely, a demand charge only affects demand in a minority of peak load episodes, although it does effectively reduce power requirements during those events, as discussed later. Demand-aware and TOU electricity tariffs can therefore encourage demand response to serve alternative system-level goals: peak shaving or load shifting, respectively.

**Figure 2:**
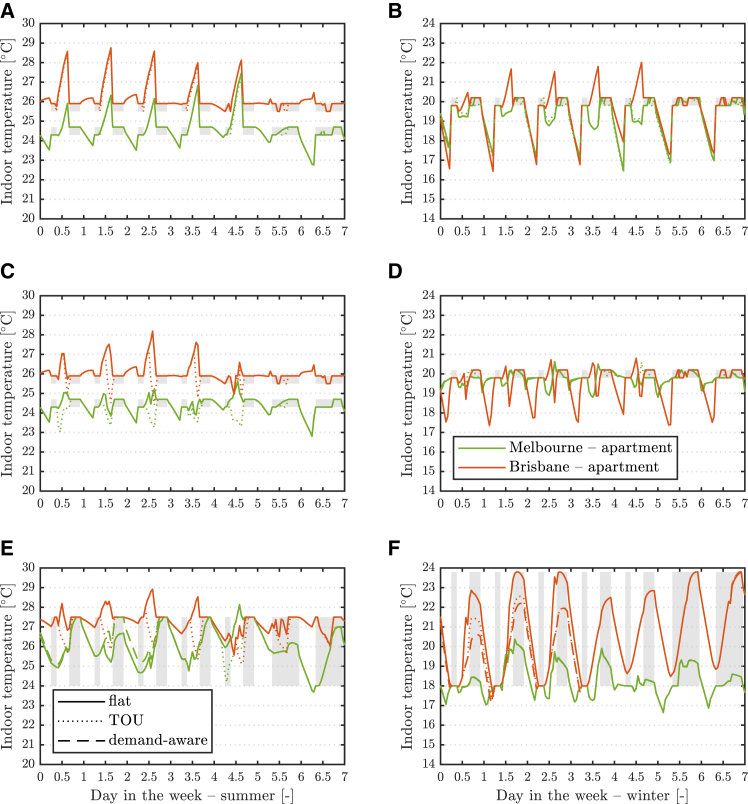
Indoor temperatures with electrification and DER for various electricity tariffs and TCM strategies Building indoor temperature evolution for one week in summer (left) and winter (right) at two sites with full electrification and DER and for three electricity tariffs with tight setpoint and no pre-heating/cooling (top), tight setpoint with pre-heating/cooling (middle), and loose setpoint with pre-heating/cooling (bottom). Only key results for new apartments are shown. The shaded regions identify the thermostat setpoint range and timing. Note the different scales for the y axis.

Similar temperature trends are observed for selected cases with full electrification and DER in Figure 2. See Figures S3 and S4 for the full suite of results. Now, houses make use of rooftop PV for building pre-heating/cooling in the middle of the day, regardless of the electricity tariff. Interestingly, apartments with limited roof space still need a TOU tariff to encourage pre-cooling. Electrification of heating plus rooftop PV may also result in higher indoor winter temperatures in Melbourne under the loose thermostat setpoint (Figure 2F).

Occupants’ thermal comfort can therefore be maintained under all the TCM strategies considered, *together* with their allowing for more flexible demand and limiting the required size of some of the installed equipment. The associated capital, energy, and emission savings are now detailed in the following sections.

### Harnessing thermal comfort management for cost and emission reductions

**Figure 3:**
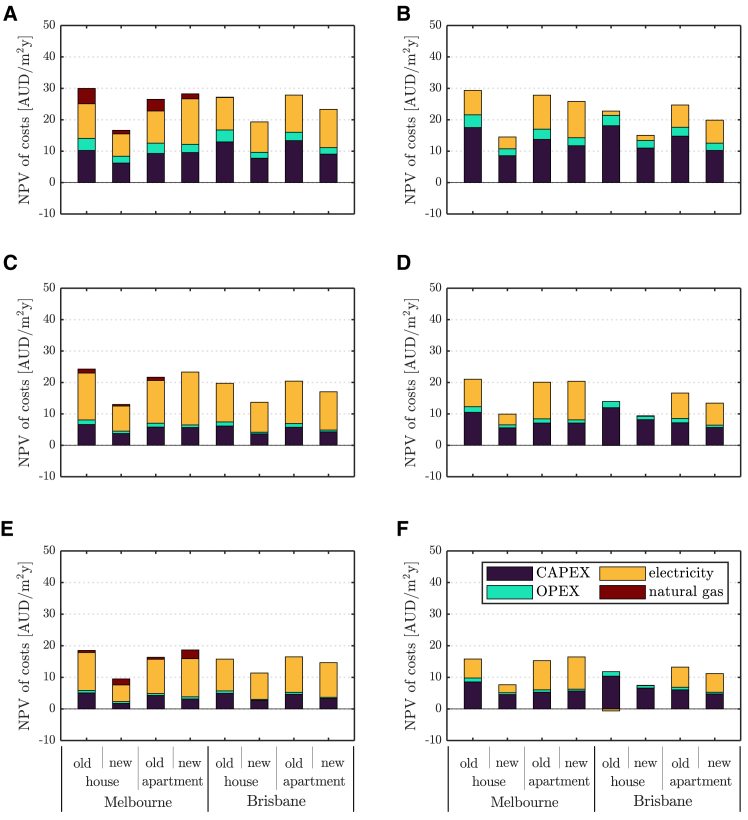
Costs without DER for various TCM strategies Annualised net present value (NPV) of total cost breakdown for cases without DER (left) and full electrification and DER (right), and a demand-aware tariff, various sites and building types, with a tight setpoint without pre-heating/cooling (top), a tight setpoint with pre-heating/cooling (middle), and a loose setpoint with pre-heating/cooling (bottom).

Figure 3 shows the annual cost to the homeowner from supplying household energy demand in some of the scenarios explored with a demand-aware tariff, since Figures S5 and S6 demonstrate a rather limited effect of tariff structures on total costs. A demand-aware tariff was nonetheless found to promote a shift away from natural gas toward more electricity consumption in dual-fuel dwellings and, importantly, often allows energy supply at or near the lowest cost (see, for instance, Figure S5). Significant cost variations are observed across the houses considered and are mainly due to.

(1) adopting more flexible TCM strategies;
(2) enabling DER, particularly with a fully electrified energy supply; and
(3) differences between buildings of different vintage.

Building pre-heating/cooling and a looser thermostat setpoint both yield significant cost reductions across climates and building types. Savings of 20–40% are predicted for both cases without DER and with full electrification and DER (Table S1). Moreover, savings are typically larger with full electrification and DER, where they mainly stem from lower capital expenditure. When pre-heating/cooling is enabled (Figures 3D and 3F), annualized capital costs for full electrification and DER are comparable to those estimated in cases without DER and an inflexible TCM strategy (Figure 3A). Otherwise, full electrification and DER adoption with tight setpoint and without pre-heating/cooling (Figure 3B) could require 38% higher upfront investment than the same case without DER (Figure 3A), on average, across the buildings and sites considered, and up to 70% higher investment for the old house in Melbourne. This shows that suitable TCM strategies could alleviate the investment burden of DER adoption, thus making building electrification significantly more affordable.

Building pre-heating/cooling primarily avoids capital costs. It enables the downsizing of some of the energy supply equipment that now operates at reduced load. For instance, the rating of the gas boiler supplying the old house in Melbourne is reduced from 18 to 8 kW_th_. The discussed higher demand flexibility then promotes additional savings from a better response to price signals from the electricity tariffs. On the other hand, a looser thermostat setpoint further reduces running costs by relaxing the time over which equipment should be operated with the allowed temperature swings. In this, TCM appears superior to demand response strategies relying on additional flexible technologies, e.g., thermal energy storage, which entail higher costs for the occupant.^33^ Lower savings are estimated when operation only is optimized for the selected TCM strategy (Table S1). This underscores the value of accounting for TCM-induced flexibility also at the design stage, when the capacities of DER and appliances can be optimized. Yet, sizing practices and climatic design conditions in building standards aim at ensuring peak heating/cooling loads are satisfied and currently do not allow for similar considerations.

**Figure 4:**
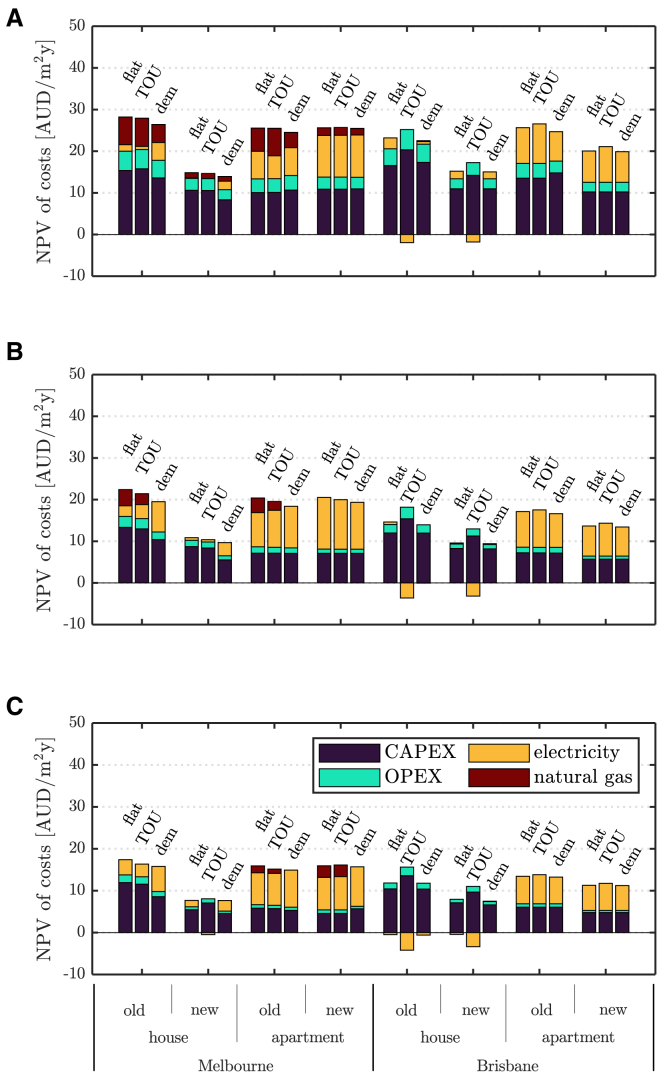
Costs with electrification and DER for various electricity tariffs and TCM strategies Annualized net present value (NPV) of total costs breakdown for cases with DER and various electricity tariffs, sites, and building types, with tight setpoint without pre-heating/cooling (top), tight setpoint with pre-heating/cooling (middle), and loose setpoint with pre-heating/cooling (bottom).

These and the results in Figure 4, where the solver was free to select between dual-fuel household supply and full electrification, also suggest that flexible TCM strategies in conjunction with DER can be a viable means of reducing GHG emissions while containing total costs. Reduced reliance of buildings on gas was also observed in some of the temperate climate cases when adopting flexible TCM strategies. When both dual-fuel household energy supply and full electrification are allowed, a loose thermostat setpoint with pre-heating/cooling (Figure 4C) makes electrification and DER the least-cost energy supply solution for the investigated detached houses in Melbourne. Household gas demand is nil for all tariff structures in this case. Furthermore, gas usage is significantly reduced when only pre-heating/cooling is allowed (Figure 4B). In this case, cost savings of dual-fuel households compared to their fully electrified counterparts are never larger than 7%, so even a small relaxation of the thermostat setpoint may then provide a sufficient cost reduction for the homeowner to electrify.

Finally, the value of new building practices targeting higher energy efficiency is also apparent in Figures 3 and 4, particularly for detached houses. Similar annual savings of up to about 12 AUD/m^2^ are found in temperate climates, with minor variations by tariff. However, in sub-tropical climates, improved thermal insulation can lead to an undesired increase in cooling load when large solar and internal gains occur, such that the value of higher energy efficiency looks to be about half of that in temperate climates.

**Figure 5:**
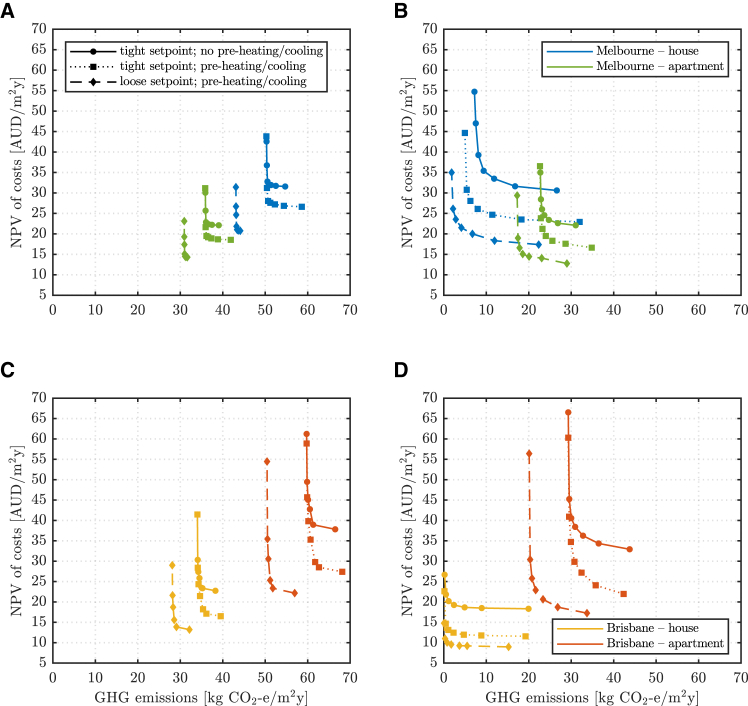
Cost-emissions trade-offs for various TCM strategies Trade-offs between annualized net present value (NPV) of total costs and yearly GHG emissions per unit building floor surface for cases without DER (left) and full electrification and DER (right) and a flat electricity tariff, for two old building types in Melbourne (top) and Brisbane (bottom) and alternative TCM strategies (solid, dotted and dashed lines).

Alternative TCM strategies can also enable significant GHG emissions abatement, with associated trade-offs on the costs to the homeowner as shown in Figure 5. Across cases, emission reductions can be initially achieved with operational changes and a limited rise in running costs. Further abatement then requires a different sizing and selection of the energy technology mix, with a larger cost increase and progressively lower emission reductions. All cases eventually reach a maximum level of abatement that depends on the technologies allowed, the building characteristics, and the local electricity generation mix. Electricity tariffs have minor effects on GHG emissions. Instead, a looser thermostat setpoint can directly result in lower emissions by reducing energy consumption, which increases the maximum level of abatement achievable across cases.

Without investment in DER (Figures 5A and 5C) and considering the current grid generation mix, a reduction in household GHG emissions by more than 10 kg-CO_2_e/m^2^ per annum could be achieved solely via a relaxation in temperature setpoint. This is a roughly 20% reduction in GHG emissions at no cost. On the contrary, building pre-heating/cooling primarily affects costs rather than emissions, as shown in Figures 3 and 4. Compared to the TCM strategy with a tight thermostat setpoint and no pre-heating/cooling, it allows reaching a given level of emissions with lower associated costs, but little GHG impact. Indeed, GHG emissions when pre-heating/cooling is enabled could be even slightly higher in some cases, as more energy is consumed. A slight GHG emissions increase is observed in Melbourne due to the coal-dominated generation mix, which causes higher emissions as a larger portion of household thermal demand is electrified. As the decarbonization of the electricity system progresses, GHG abatement may also be reached when building pre-heating/cooling is allowed. Importantly, however, it is system-level changes – and not the TCM strategy alone – that lead to this outcome.

In stark contrast, the results obtained with full electrification and DER (Figures 5B and 5D) show significantly lower levels of GHG emissions and only minor changes in total costs. In line with previous findings,^34^ this illustrates the primary role that DER could play in enabling emissions abatement from buildings. Additionally, this work demonstrates how a suitable TCM strategy could be chosen to support this abatement, for example, by maintaining total costs while reducing emissions via greater flexibility and the use of more DER. On one hand, allowing building pre-heating/cooling is shown to favor electrification and DER adoption. An increased electricity consumption makes DER more economically attractive by decreasing their time to pay back and reducing homeowner costs. On the other hand, a different TCM strategy with a looser thermostat setpoint is proven to reduce emissions independently from DER. It could be a preferable alternative to investing in DER, as well as suitable for apartments with limited roof availability. Furthermore, a loose thermostat setpoint appears necessary to reach an almost complete cut in GHG emissions from old buildings. The Pareto fronts for new buildings are reported in Figure S7.

### Implications on electricity peak loads

**Figure 6:**
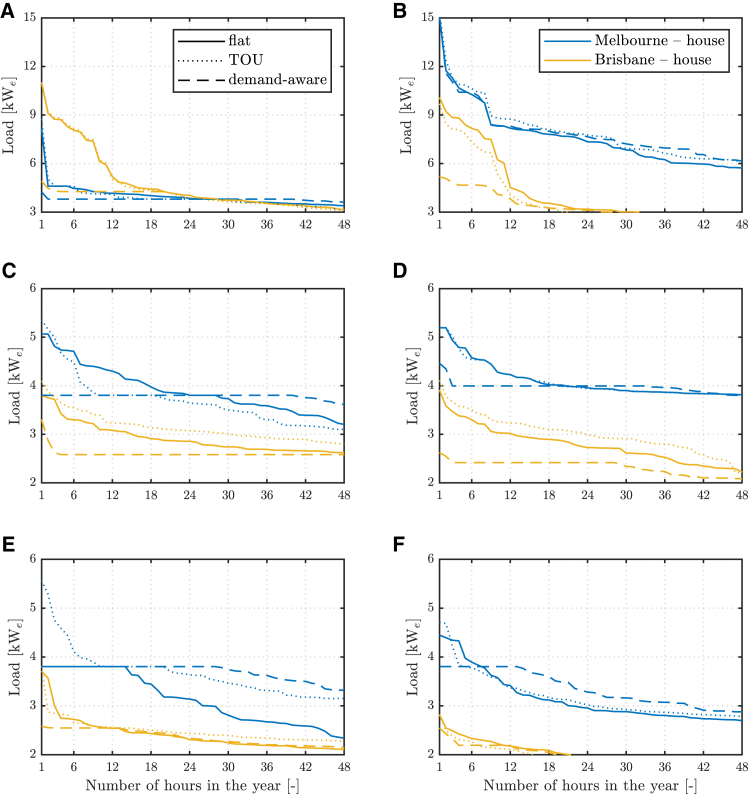
Electricity loads for various electricity tariffs and TCM strategies Electricity load duration curves of the highest peak loads for cases without DER (left) and full electrification and DER (right), various sites and the three electricity tariffs with tight setpoint without pre-heating/cooling (top), tight setpoint with pre-heating/cooling (middle), and loose setpoint with pre-heating/cooling (bottom row). Only key results for old houses are shown.

Shifting to system-level implications, Figure 6 shows how suitable tariffs and TCM strategies can limit the peak loads requested by individual dwellings. These loads are supplied by an upstream electricity distribution network, whose costs currently account for about one-third of what customers pay for electricity in most Australian jurisdictions.^35^

A demand-aware tariff is effective in reducing electricity peaks across climates and for both dual-fuel (Figures 6A–6C and 6E) and fully electrified dwellings (Figures 6B–6D and 6F). It also slightly decreases homeowner costs, as previously noted, thus appearing as a potential pathway to achieve cost-effective peak load reductions from single households. Previous work suggests these outcomes are also robust against the selected tariff values, so long as a demand charge is considered.^20^ Conversely, other studies find demand charges can be less effective for building aggregations as they only target rare peak events, which occur at different times.^36^ Demand-aware tariffs also result in overall higher electricity consumption, due to reduced prices outside peak events, as can be observed in Figure 6, as well as in the full load duration curves in Figures S8 and S10. This increase in electricity consumption is observed particularly for cases with full electrification and DER, and houses in Melbourne can reach one and a half times the predicted consumption under a flat tariff. Nonetheless, this increased load is mainly shifted toward hours of low demand, which confirms findings from the analysis of 10-year, real-life electric load data in^36^ and could help, along with other measures, to avoid renewable curtailment in future grids, thus mitigating a reported drawback of increased building efficiency.^37^

Time-of-use tariffs are found to be ineffective in reducing high loads that occur outside peak windows and, in some cases, can even lead to an uptick in power loads. Previous work on time-based electricity pricing also anticipates similar load rebounds.^28^^,^^38^ Moreover, although the definition of peak windows in TOU tariffs often aligns with high electricity demands in the evenings, peak thermal demand does not always coincide with windows of high electricity prices. This could further hinder the effectiveness of TOU tariffs in reducing loads as heating becomes electrified, especially in colder climates (Melbourne), where electric heaters are chosen as the cheapest technology to supply morning heating peaks or cold spells throughout the day. Another effect of time-based pricing not investigated here is a loss of end users’ demand diversity with potential network impact.^38^

TCM strategies allowing more load flexibility are shown to play a role also in reducing upstream peak load requirements. Pre-heating/cooling already enables significant peak reductions across old buildings, which confirms the outcomes from studies on home pre-cooling only.^20^ Compounded by a demand-aware tariff, peak loads can be more than halved. A looser thermostat setpoint can bring further, albeit marginal, reductions. Minor differences in the scope of peak load reduction via electricity tariffs or TCM emerge across building types and, more importantly, vintage, due to initially lower peaks for the new buildings assessed. Notably, a similar compounding effect of a TCM strategy allowing building pre-heating/cooling and a demand-aware tariff is observed, with peak load reductions of up to about 20% projected for new buildings, increasing to about 25% when the temperature setpoint is loosened. The load duration curves in these cases are reported in Figures S10 and S11. A 1°C–3°C setback on heater setpoint following building pre-heating was indeed tested in real-life trials and conducive to 0.2–0.65 kW_e_ lower household demand at peak times, with adequate comfort levels for participants.^39^

Finally, previous work suggests that electrification could significantly increase aggregated peak loads.^40^ Findings from this work for full electrification and DER (Figure 6B) also show this, particularly for the detached house in Melbourne with the tight setpoint. However, these results also demonstrate that the strategies investigated can reduce peak loads down to similar values to those predicted for dual-fuel dwellings. Thus, the electrification of building heating and cooling needs, when properly orchestrated through suitable electricity tariffs, TCM strategies, or other means, need not result in significant peak load increases. On the contrary, resulting peak loads could even be lower. This echoes observations from demand-response trials on 14 fully electric dwellings in the UK, where a 21% reduction in low-voltage feeder power during 2-h turn-down events was reported, with little change to occupants’ comfort.^41^ Direct benefits from avoided or contained capacity augmentation are then expected for distribution network operators and, ultimately, consumers via contained network charges.

### Aggregate peak load projections and their mitigation

**Figure 7:**
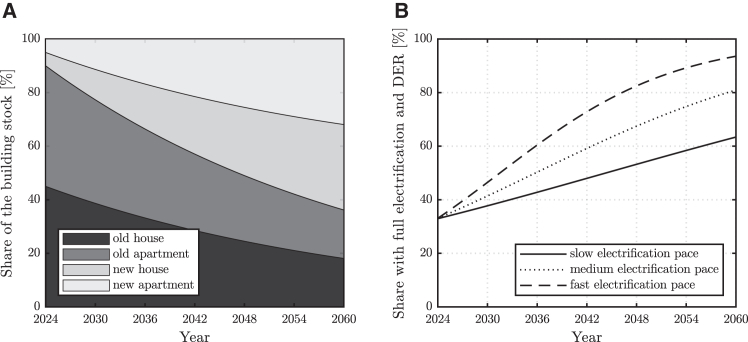
Modeled building stock projections Evolution of the modeled building stock served by a medium-density feeder with a 2.5% per annum building renovation rate and various paces of electrification and DER uptake. Building types and vintage (left) and their share with full electrification and DER (right).

To conclude, aggregate peak load projections and potential mitigation at a model electricity distribution feeder are discussed in the following, considering various evolutions of the residential building stock. This analysis starts from the peak load results obtained for progressively higher shares of household electrification and DER in Table 2. These are then mapped over time using logistic S-curves that reflect a plausible deployment of electric household appliances in Australia^42^ and an evolving ratio of old to new dwellings which mimics a renovation rate of 2.5% of old buildings per annum.^43^ An example of the building stock evolution served by a medium-density distribution feeder is reported in Figure 7. Readers are directed to Table S5 for a detailed definition of the assumed combinations of building types and vintage in each case. Based on these assumptions, the aggregated peak load mitigation potential of the different measures investigated in this work is then assessed.

**Table 2:** Projected peak load values for various shares of electrification and DER at two sites, for a feeder supplying residential customers in areas with various population densities and thus different building combinations

Electrification and DER share [%]	Melbourne [p.u.]			Brisbane [p.u.]		
	Low-density feeder	Med-density feeder	High-density feeder	Low-density feeder	Med-density feeder	High-density feeder
0	1.00	1.00	1.00	1.00	1.00	1.00
20	1.16	1.14	1.13	0.98	0.99	0.99
40	1.32	1.29	1.26	0.97	0.97	0.98
60	1.48	1.43	1.39	0.95	0.96	0.97
80	1.64	1.58	1.52	0.94	0.95	0.96
100	1.80	1.72	1.65	0.92	0.94	0.95

See Table S5 for the assumed combinations of building types and vintage in the various density areas considered.

**Figure 8:**
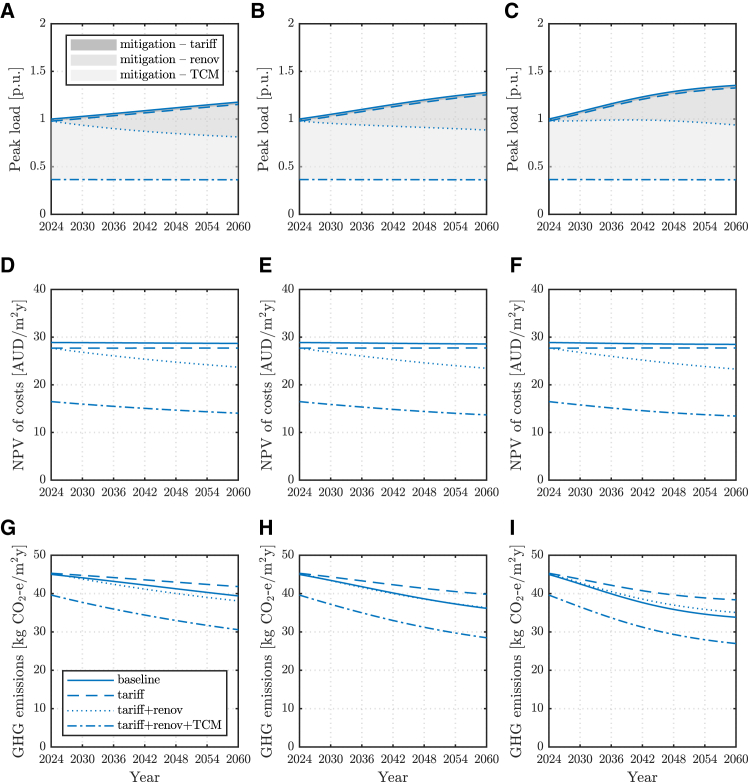
Peak load, cost and emissions projections and mitigation potential in Melbourne Aggregate peak load projections and mitigation potential of various strategies (top) average household annualized NPV of total costs (middle) and GHG emissions (bottom) for a medium-density feeder in Melbourne for slow (left), medium (center) and fast (right) paces of electrification and DER, and a 2.5% per annum renovation of the building stock.

**Figure 9:**
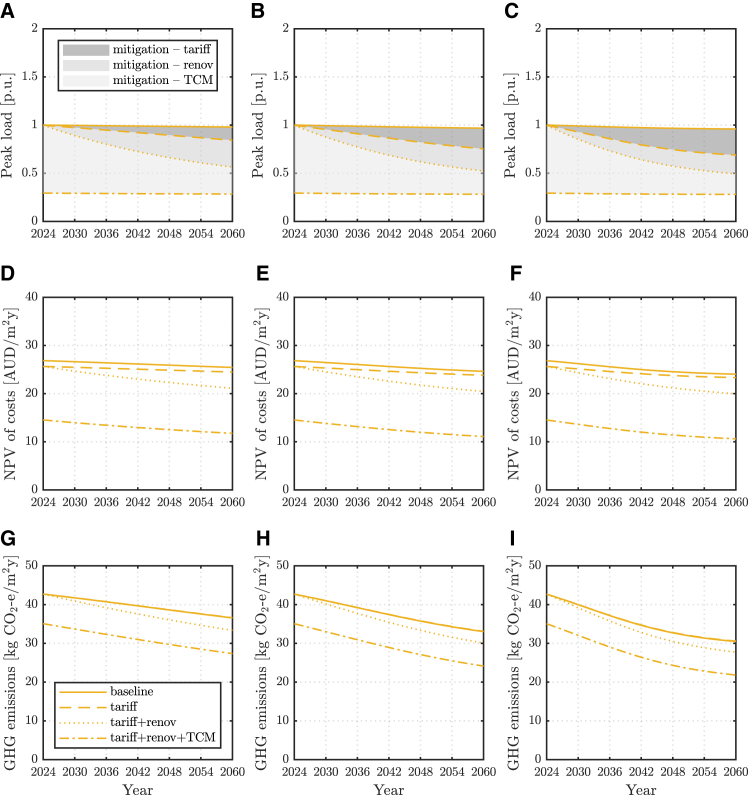
Peak load, cost and emissions projections and mitigation potential in Brisbane Aggregate peak load projections and mitigation potential of various strategies (top) average household annualized NPV of total costs (middle) and GHG emissions (bottom) for a medium-density feeder in Brisbane for slow (left), medium (center) and fast (right) paces of electrification and DER, and a 2.5% per annum renovation of the building stock.

Figures 8 and 9 illustrate, in solid lines, the projected evolution of aggregated peak loads at the distribution feeder level in each of the considered cases. Other line styles then show the aggregated peak loads as a progressive combination of the strategies investigated in this work is enacted. Shaded areas, therefore, represent the peak load mitigation potential estimated for each strategy. The resulting average customer cost and GHG emissions per unit floor surface from each strategy are also reported, respectively, on the middle and bottom rows. Results for a low- and a high-density feeder are reported in Figures S12–S14.

Aside from electric vehicle uptake, which is not considered here, any increase in peak load is to be expected mainly from the electrification of the thermal demand that is currently supplied by gas. Aggregated peak load projections are therefore higher in temperate climates (Figures 8A–8C) and for larger shares of detached houses, given these have higher thermal needs (Table 2). In sites where most thermal needs are already electrified or small (e.g., in sub-tropical climates, Figures 9A–9C), DER uptake will aid electricity supply from the grid and could indeed lower the requested peak load.^40^

Demand-aware tariffs are more effective in reducing loads for higher shares of electrification and DER uptake (Figure 6). Accordingly, they could play a bigger role in sub-tropical climates, and as the share of fully electrified dwellings increases. However, without the flexibility of a looser thermostat setpoint and/or a thermal delivery schedule allowing for building pre-heating/cooling, their impact on aggregated peak load reduction is very limited, particularly in temperate climates with large morning heating peaks.

Building renovation targeting higher energy efficiency and the use of suitable TCM strategies has the greatest potential for aggregate peak load reduction. These strategies also yield further social benefits as household cost and GHG emissions reduction, through the effects discussed in previous sections. The breadth and depth of adoption of these measures depend on local policies and are subject to customers' decisions, but co-deployment can yield the largest load impact.^44^ Also, it is noted that higher building stock retrofit rates of 5% – and even up to 10% – per annum have been suggested.^43^ These involve much more aggressive building renovations than what is currently registered across many economies.^45^^,^^46^ Nonetheless, this work finds a substantial peak load mitigation potential also at a more moderate 2.5% renovation rate. Therefore, even under segregated or limited intervention, building renovation and adoption of TCM strategies should be pursued.

Furthermore, while renovation may be less attractive for the involved financing, TCM has little or no cost, and on the contrary, yields savings to the homeowner and reduced system GHG emissions (Figures 8 and 9, middle and bottom rows). It therefore appears as a viable measure to contain system-level burden independent from ambitious building renovation rates to be realized in real practice and/or for extended building lives due to insufficient building up of new dwellings. Benefits are shown to potentially more than offset the peak load increase due to the electrification of thermal demand,^47^ translating into power security,^48^ limited strain on electricity distribution networks, and cost-saving potential.^49^ For instance, a 5% reduction in peak capacity would lower distribution charges and result in a 1.4–1.6% decrease in the retail price, for the current average electricity bill. Results in Figures 8 and 9 suggest peak load reductions could be significantly larger.

Significant untapped potential is demonstrated across building types and vintage, particularly in the use of TCM strategies to reduce homeowner total costs and yearly building GHG emissions and favor DER adoption. On the other hand, the much-debated electricity tariff design emerges as a weaker driver of aggregated system benefits, especially in temperate climates, and its scope is limited in the absence of a looser thermal setpoint. Of course, the realization of system benefits will ultimately be subject to the customer’s acceptance and adoption of the measures discussed. Supporting policies could, for instance, subsidize the installation of smart home energy management systems^50^ and promote flexibility in occupants’ approach to TCM by informing on the financial benefits associated. These results indeed demonstrate both system and customer gains, thus anticipating room for this flexibility to be rewarded. A suite of incentives – e.g., low-interest loans, grants, tax relief – that appeal to different cohorts^51^ could then be devised to broaden participation.

### Conclusion

This work examined the potential of combining electricity tariffs, thermal comfort management, and building practices for several sites and building types as a means to reduce household energy costs, GHG emissions, and peak loads on electricity distribution networks. Each of these measures was shown to have a different and potentially significant role.

• While thermal management allows for a looser thermostat setpoint has energy and emissions savings potential on its own, the use of building pre-heating/cooling enables larger peak loads and cost reductions. These can help reduce the capital cost burden of DER and other capital-intensive, low-carbon energy supply options such as heat pumps. These strategies are a largely untapped way to enable building decarbonization while increasing affordability across climates and building types.
• Electricity tariffs that include a demand charge cause cooling peak load reductions without increasing costs to homeowners, provided they respond in a close-to-optimal manner. This is expected to drive further savings to network operators and likely reduce customers’ bills, especially in warm climates, where it could therefore realize a significant social benefit.
• Compared to other peak load mitigation options, building renovation for higher energy efficiency has variable economic and emissions-saving potential depending on the building type and local climate. The combined use of a demand-aware tariff and TCM can lead to similar, if not lower, homeowner costs, even when upfront renovation costs are ignored.

A roughly 20% reduction in building GHG emissions across climates and building types appears to be achievable at no cost and without compromising occupant’s thermal comfort, only by relaxing thermostat setpoints. This would further enable containment, and even avoid, anticipated peak load increases and operability issues that building electrification may impose on electricity networks. Thus, flexible thermal comfort management emerges as an enabler of electrification that facilitates energy system decarbonization. It is therefore recommended that future residential building decarbonization policies include strategies to promote the adoption of flexible thermal comfort management alongside the more traditional interventions mandated in current building standards.

### Limitations of the study

This study, as with any modeling exercise, is affected by uncertainty. First, the projected results assume optimal responses of households to different price signals, which may not reflect several consumers’ behaviors, with price unresponsiveness,^52^ distrust of automation,^53^ or the impossibility of deferring some loads,^54^ to name a few. Second, although trials depict ASHRAE’s comfort range as rather conservative,^55^ acceptability limits are of course, subjective and dictated by users’ pref. 56. Some cohorts may not be willing or even flexible to adjust thermostat setpoints.^57^ Third, this work compares a limited number of cases combining electricity tariffs, building types, and climates. We approached such limitations by selecting tariff structures which, when combined and/or with adjusted parameters, span most current offerings. Similarly, building archetypes span a majority of dwellings, and physics-based models tie loads to local climate, thus limiting specificity. We, therefore, emphasize the value of these results in linking various drivers to the expected household response and, through this, highlighting the *potential* of the investigated strategies to support building decarbonization. Of course, the presented cost, energy and emission figures are case-dependent and will differ in practice. Yet, given the demonstrated breadth of this *potential*, the proposed strategies should remain effective with more limited adoption/response, and their wider use in other contexts may also be plausible.

## Resource availability

### Lead contact

Further information and requests for resources and reagents should be directed to and will be fulfilled by the Lead Contact (a.vecchi@unimelb.edu.au).

### Materials availability

This study did not generate new unique materials.

### Data and code availability

• The sources of the datasets supporting the current study are presented in the key resources table and the method details sections.
• Requests for the data and code that support the findings of this study should be directed to the lead contact.
• Any additional information required to reanalyze the data reported in this article or reproduce the results is available from the lead contact upon request.

## Author contributions

Andrea Vecchi: conceptualization, methodology, formal analysis, writing - original draft, writing - review and editing, and visualization; Michael John Brear: supervision, conceptualization, writing - review and editing, and funding acquisition.

## Declaration of interests

The authors declare no competing interests.

## STAR★Methods

### Key resources table

**Table:** 

REAGENT or RESOURCE	SOURCE	IDENTIFIER
**Deposited data**		

Hourly weather traces	Sengupta et al.^58^	https://nsrdb.nrel.gov/
Archetypal building data	CSIRO^59^	https://ahd.csiro.au/
Residential electricity tariff structures and details	AER^60^^,^^61^	https://www.aer.gov.au/publications/reports/performance/state-energy-market-2021 https://www.aer.gov.au/industry/networks/pricing-proposals-and-tariff-variations
Recommended thermal comfort conditions	ANSI/ASHRAE^62^	https://webstore.ansi.org/standards/ASHRAE/ansiashraestandard552023
Results from this study	This paper and companion repository	https://figshare.com/s/85adc27fb68946dd1655

**Software and algorithms**		

Python 3.10	Python Software Foundation^63^	https://www.python.org/
Gurobi 11.0.0	Gurobi Optimization LLC^64^	https://www.gurobi.com/
MATLAB R2023b	MathWorks^65^	https://au.mathworks.com/
Detailed mathematical model formulation	Vecchi et al.^34^	https://doi.org/10.1016/j.jclepro.2024.141465

### Method details

A mixed integer linear programming (MILP) model was developed to optimise the technology mix, capacity and hourly schedule, such that household energy demands are supplied at least cost to the homeowner. Hereafter, the salient features of this modeling are outlined; a link to the full mathematical model formulation is reported in the key resources table. Figure S16 presents the energy supply and storage technologies considered in the modeling and the superstructure for their interaction to meet household electricity, heating, cooling and domestic hot water demand.

#### Optimal design and operation of building energy supply

As detailed in a previous work,^34^ the model minimises the net present value of technology investment ($Z_{i,j}^{inv}$) and running costs ($Z_{i,j}^{run}$) over the study time frame, including the capital expenditure (CAPEX), fixed and variable operating costs (OPEX), salvage value and energy purchase cost for several supply (i) and storage (j) technologies (see Figure S12), which depend on their capacity ($X_{i,j}$) and schedule ($x_{i,j}$). Discounting of future cash flows in year y to the present was performed using a 5% discount rate r^34^.

(Equation 1) $$\min_{X_{i,j},x_{i,j}}\;NPV=\sum_{i,j}Z_{i,j}^{inv}\left(X_{i,j}\right)+\sum_{y}\frac{\sum_{d,t}Z_{d,t}^{run}\left(x_{i,j}\right)}{{\left(1+r\right)}^{y}}$$

Technical constraints describing the energy conversion and storage efficiency of each modeled technology were included, which consider temperature dependencies of the coefficient of performance,^66^^,^^67^^,^^68^ instantaneous solar irradiation on rooftop photovoltaics^69^ and solar thermal collectors,^26^ energy storage standing losses and the charge/discharge efficiency.^70^ These constraints were complemented by limitations on the roof space availability for solar panel installation by building type.

Energy conservation in the electric, heating, cooling and domestic hot water supply systems was imposed separately, and individual energy demands must be satisfied at any time. Electric appliance demand profiles were based on real-life metering.^71^ Domestic hot water needs reflecting the recommended daily value of 40 L per person and the number of occupants per unit floor surface^72^ were uniformly spread across a 3-h (6:00-7:00 and 17:00-19:00) and a 6-h window (12:00-18:00), respectively, for weekdays and weekends. Finally, heating and cooling demands arise in the model from the need to maintain the household indoor temperature within the comfort range dictated by the selected TCM strategy and follow both occupancy and weather conditions, as described hereafter.

#### Dynamic building model

A lumped, 4R1C model was developed to capture building temperature ($\theta_{d,t}^{BDG,1}$ and $\theta_{d,t}^{BDG,2}$) dynamics, simplifying the approach from standard ISO 13790^73^ to account for building fabric, windows and air tightness through three separate thermal resistances, respectively $R^{th,2}$, $R^{th,3}$ and $R^{th,4}$. The resistance $R^{th,1}$ determines the convective and radiative heat transfer between the indoor volume and the building surface, and $C^{th}$ is the thermal capacity of the building. Suitably discretised over the study timestep Δt, the 4R1C model results in a set of linear equations

(Equation 2) $$\frac{\theta_{d,t}^{BDG,1}-\theta_{d,t}^{BDG,2}}{R^{th,1}}+\frac{\theta_{d,t}^{BDG,1}-\theta_{d,t-1}^{amb}}{R^{th,4}}=q_{d,t-1}^{BDG,1}+\eta^{HC}\left(h_{d,t-1}^{BDG}-c_{d,t-1}^{BDG}\right)$$

(Equation 3) $$C^{th}\frac{\theta_{d,t}^{BDG,2}-\theta_{d,t-1}^{BDG,2}}{\Delta t}+\frac{\theta_{d,t}^{BDG,2}-\theta_{d,t-1}^{amb}}{R^{th,2}}+\frac{\theta_{d,t}^{BDG,2}-\theta_{d,t-1}^{amb}}{R^{th,3}}=\frac{\theta_{d,t}^{BDG,1}-\theta_{d,t}^{BDG,2}}{R^{th,1}}+q_{d,t-1}^{BDG,2}$$

where $h_{d,t}^{BDG}$ and $c_{d,t}^{BDG}$ represent, respectively, the aggregated heating and cooling supplied by the installed technologies, with delivery efficiency $\eta^{HC}$. Internal gains $q_{d,t}^{BDG,1}$ were computed from tabulated hourly values from occupants and appliances in residential buildings, per unit floor surface ($A^{fl}$).^73^ These tabulated values (in W/m^2^) differ to reflect typical diurnal variations in the heat generation from building occupants and appliance use for a conventional working and a non-working day. They therefore required distinguishing between weekdays

(Equation 4) $$q_{d,t}^{BDG,1}=\{\begin{array}{ll} {1e}^{-3}\cdot \left(0.7\cdot 2+0.3\cdot 6\right)A^{fl} & if\;t<7⋁t\geq 23\\ {1e}^{-3}\cdot \left(0.7\cdot 20+0.3\cdot 1\right)A^{fl} & if\;17\leq t<23\\ {1e}^{-3}\cdot \left(0.7\cdot 8+0.3\cdot 1\right)A^{fl} & if\;7\leq t<17 \end{array}$$

and weekends.

(Equation 5) $$q_{d,t}^{BDG,1}=\left\{ \begin{array}{ll} {1e}^{-3}\cdot \left(0.7\cdot 2+0.3\cdot 6\right)A^{fl} & if\;t<7⋁t\geq 23\\ {1e}^{-3}\cdot \left(0.7\cdot 20+0.3\cdot 4\right)A^{fl} & if\;17\leq t<23\\ {1e}^{-3}\cdot \left(0.7\cdot 8+0.3\cdot 2\right)A^{fl} & if\;7\leq t<17 \end{array}\right.$$

Solar gains $q_{d,t}^{BDG,2}$ were computed from the window surface ($A^{wd}$) and frame factor ($F^{f}$), glazing solar heat gain coefficient (SHGC) and the hourly value of the incident solar irradiance $G_{d,t}$, whilst neglecting the thermal radiation toward the sky.^73^

(Equation 6) $$q_{d,t}^{BDG,2}=F_{d,t}^{sh}\text{SHGC}\left(1-F^{f}\right)A^{wd}G_{d,t}$$

Indoor blind shading was considered through the factor $F_{d,t}^{sh}$ between 18:00 and 7:00, and each time the outdoor temperature exceeded the cooling setpoint by over 2.5°C or the incident solar radiation on the glazing was more than 200 W/m2.^74^

The 4R1C model was validated against reported experimental measurements^75^ (see Figure S17; Table S2) and used in the MILP to ensure household operating temperature satisfies the conditions required by each TCM strategy considered, as either

(Equation 7) $$\theta_{d,t}^{SP}-\Delta \theta \leq \alpha \theta_{d,t}^{BDG,1}+\left(1-\alpha \right)\theta_{d,t}^{BDG,2}\leq \theta_{d,t}^{SP}+\Delta \theta$$

or

(Equation 8) $$\theta_{d,t}^{\min}\leq \alpha \theta_{d,t}^{BDG,1}+\left(1-\alpha \right)\theta_{d,t}^{BDG,2}\leq \theta_{d,t}^{\max}$$

with α=0.5.^62^

#### Model input data and selected studies

The presented optimisation was run for a full year of operation using a 1-h resolution, 20 years as study time frame, and different allowed technology mixes, with and without DER adoption and/or full electrification, considering the following data and studies.

##### Weather data

The two Australian cities of Melbourne and Brisbane were chosen as representative of, respectively, temperate and sub-tropical climates, which span roughly 90% of Australian households and almost 70% of the worldwide population.^76^ For both sites, hourly weather traces for the 2020 calendar year were sourced from NREL.^58^

##### Buildings

A total of 8 representative buildings was considered. These include an old and a new detached house and apartment for each site and are reported in Table 1. Geometric and thermophysical parameters based on average building practices by site, type and vintage, from CSIRO’s database^59^ were used as input to the dynamic building model. When benchmarked against Australian energy efficiency ratings, old buildings score 2–5 Stars and all new buildings above 6.5 Stars.

##### Electricity tariff structures

In this work, electricity is billed to end-use customers with either.

• a flat tariff;
• a time-of-use (TOU) tariff, with time-based pricing; or
• a demand-aware tariff, that includes a usage (AUD/kWh) and a demand (AUD/kW/y) charge.

These three structures, or a combination thereof, currently cover almost all residential customers in the two Australian states considered (see Table S4). Usage charges in the former two tariffs were selected to match 2021 state-averaged values^60^ and with a TOU peak window from 15:00-21:00. In the latter, demand charges are levied on the average between the 4 largest yearly load instances and were computed on a case-by-case basis to yield the same electricity bill as the flat tariff,^27^ and for a 40:60 split between demand and usage charges.^60^ Charges are reported in Table S3. A feed-in tariff of 4.9c AUD/kWh and 5.7c AUD/kWh for Melbourne and Brisbane was also considered as per current policy.^34^ The modeling framework presented could equally be adapted to represent a range of alternative electricity tariffs offered by retailers.

##### Thermal comfort management strategy

The 3 TCM strategies investigated are.

(1) Tight setpoint without pre-heating/cooling;
(2) Tight setpoint with pre-heating/cooling; and
(3) Loose setpoint with pre-heating/cooling.

In each case, indoor temperature constraints were imposed from 6:00-10:00 and 16:00-22:00 on weekdays and from 8:00-23:00 on weekends. Thermostat setpoints for the heating and cooling season were computed for each site, as per efficiency rating practices in Australia,^74^ with an allowed 0.2°C swing for tight setpoints. For the loose setpoint, temperature variations were limited within the comfort range of 18.0°C–23.8°C in winter and 24.0°C–27.5°C in summer, as recommended by ASHRAE for a 30–60% relative humidity and appropriate clothing.^62^ Finally, pre-heating/cooling was enabled where indicated by allowing thermal provision also outside thermostat setpoint windows.

#### Aggregate peak load projections

Feeder-level peak load projections were obtained by combining the optimal results from the eight buildings investigated, with and without full electrification. Low-, medium- and high-density distribution feeders were considered, with respective shares of building types and vintage reported in Table S5. Projected peak load results as a function of the share of fully electrified buildings (Table 2) were then mapped over time with logistic, S-curves that reflect slow, medium or fast paces of electrification in Australia.^42^ The initial 90:10% ratio of old to new dwellings was also assumed to evolve in time according to a renovation rate of 2.5% of the old building stock per annum. This is the least ambitious among the recommended “deep” retrofit rates by the IPCC, for building stock decarbonisation by 2050,^43^ and it is consistent with current build rates in Australia.^77^

Peak load projections are presented in this work on a *per unit* (p.u.) base, relative to initial aggregated values for 2024, acknowledging the effect of load diversity. A rigorous account of load diversity necessitates a stochastic approach^78^^,^^79^^,^^80^ which is beyond the scope of this assessment and would be necessary to be able to present meaningful, *absolute* figures. Notwithstanding an expected change in peak load value and its time of occurrence, load diversity is however not expected to significantly alter the evolution over time of the aggregate peak loads as it is presented in this work.

### Quantification and statistical analysis

There are no quantification or statistical analyses to include in this study.

## Supplemental information

Document S1. Figures S1−S17 and Tables S1–S5
