# Supplementary material for: Using electricity tariffs and thermal comfort management to promote residential energy decarbonization
Source: iScience. 2025 May 12;28(6):112631. doi: 10.1016/j.isci.2025.112631 (PMC12177180; doi:10.1016/j.isci.2025.112631)
Supplement: Document S1. Figures S1−S17 and Tables S1–S5 [file mmc1.pdf]

iScience, Volume *28*

Supplemental information

Using electricity tariffs and thermal comfort management to promote residential energy decarbonization

Andrea Vecchi and Michael John Brear

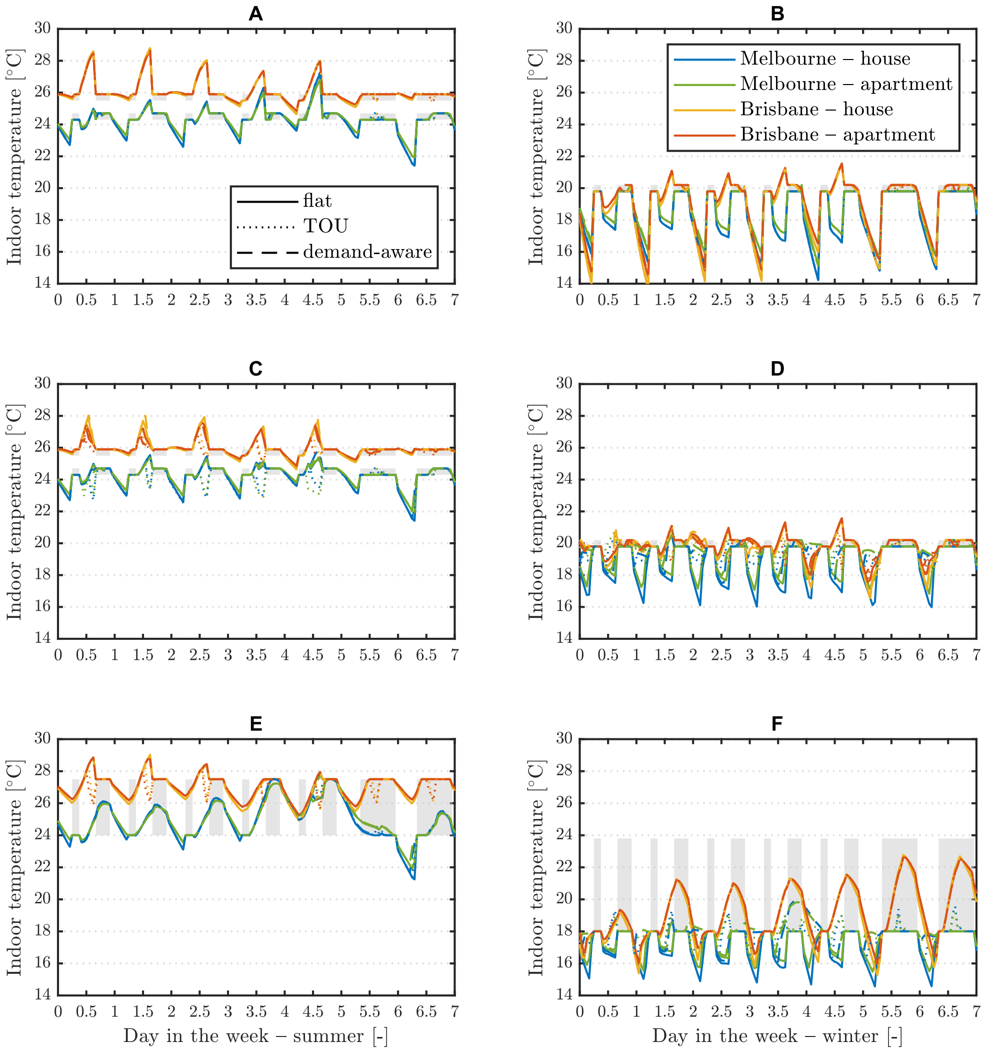

Figure S1: Building indoor temperature evolution for one week in summer (left) and winter (right) for four old building types at two sites without DER and for three electricity tariffs with tight setpoint and no pre-heating/cooling (top), tight setpoint with pre-heating/cooling (middle), and loose setpoint with pre-heating/cooling (bottom). The shaded regions identify the thermostat setpoint range and timing.

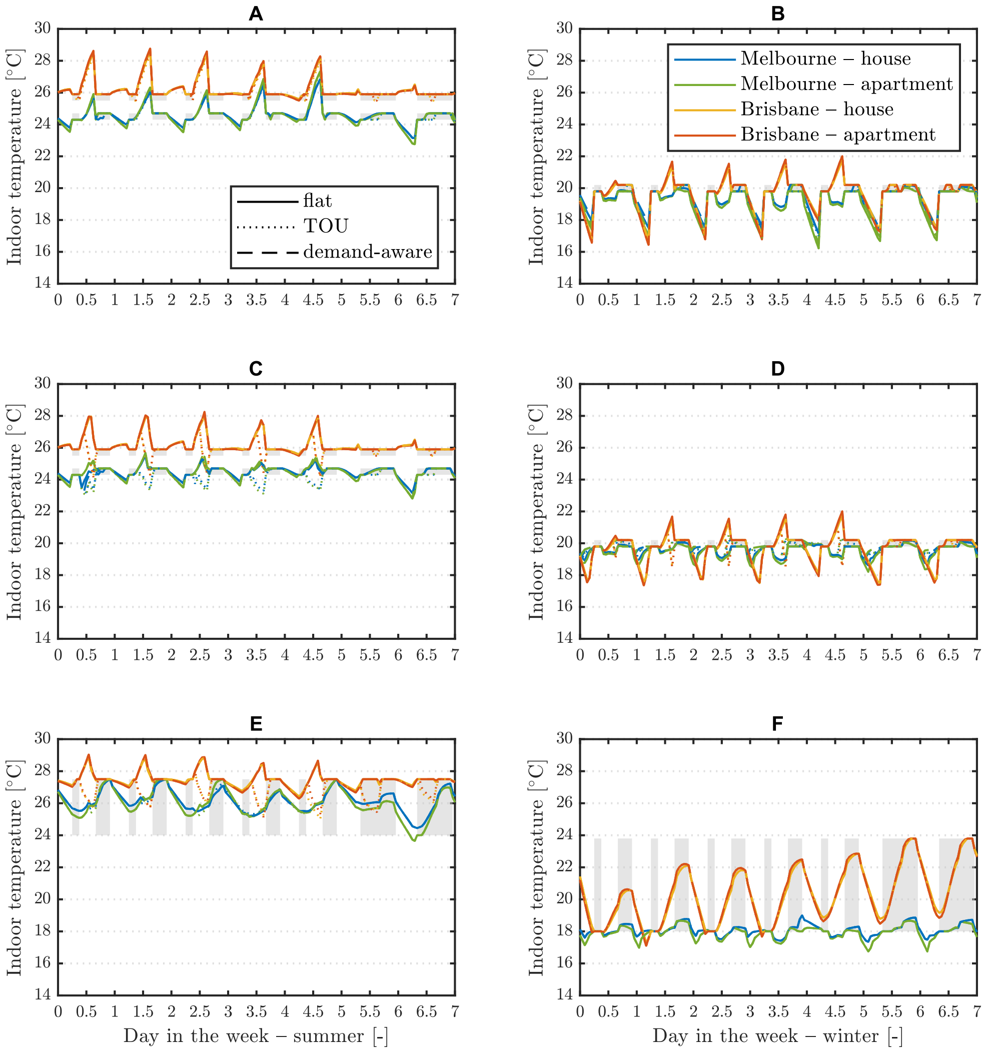

Figure S2: Building indoor temperature evolution for one week in summer (left) and winter (right) for four new building types at two sites without DER and for three electricity tariffs with tight setpoint and no pre-heating/cooling (top), tight setpoint with pre-heating/cooling (middle), and loose setpoint with pre-heating/cooling (bottom). The shaded regions identify the thermostat setpoint range and timing.

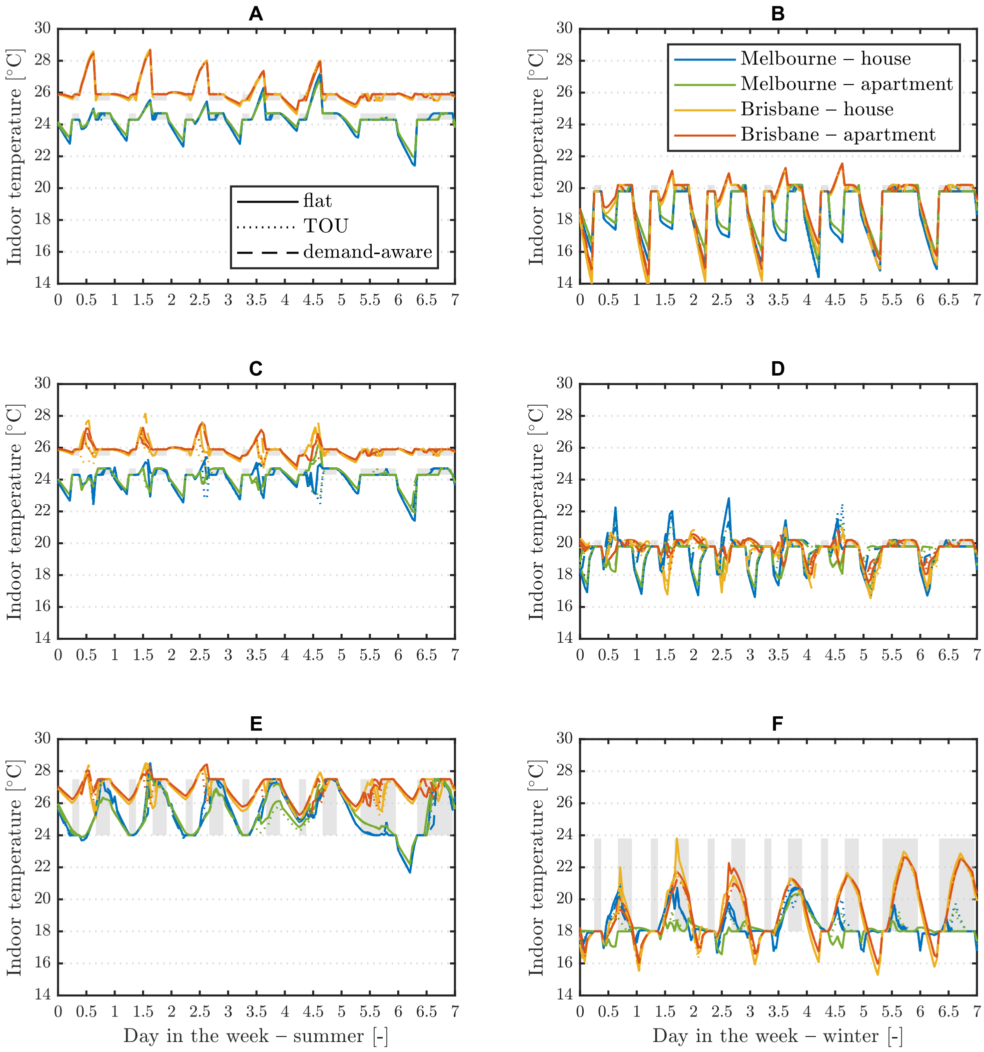

Figure S3: Building indoor temperature evolution for one week in summer (left) and winter (right) for four old building types at two sites with full electrification and DER and for three electricity tariffs with tight setpoint and no pre-heating/cooling (top), tight setpoint with pre-heating/cooling (middle), and loose setpoint with pre-heating/cooling (bottom). The shaded regions identify the thermostat setpoint range and timing.

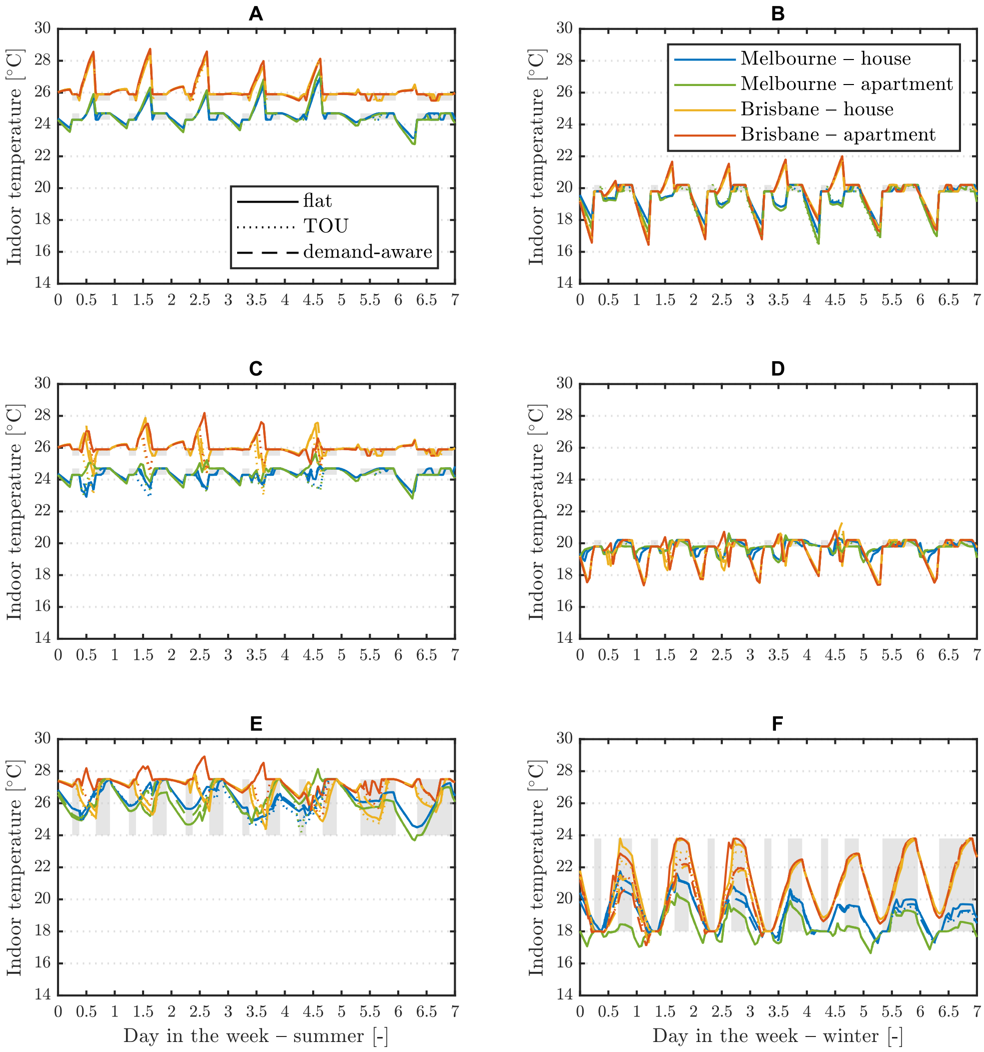

Figure S4: Building indoor temperature evolution for one week in summer (left) and winter (right) for four new building types at two sites with full electrification and DER and for three electricity tariffs with tight setpoint and no pre-heating/cooling (top), tight setpoint with pre-heating/cooling (middle), and loose setpoint with pre-heating/cooling (bottom). The shaded regions identify the thermostat setpoint range and timing.

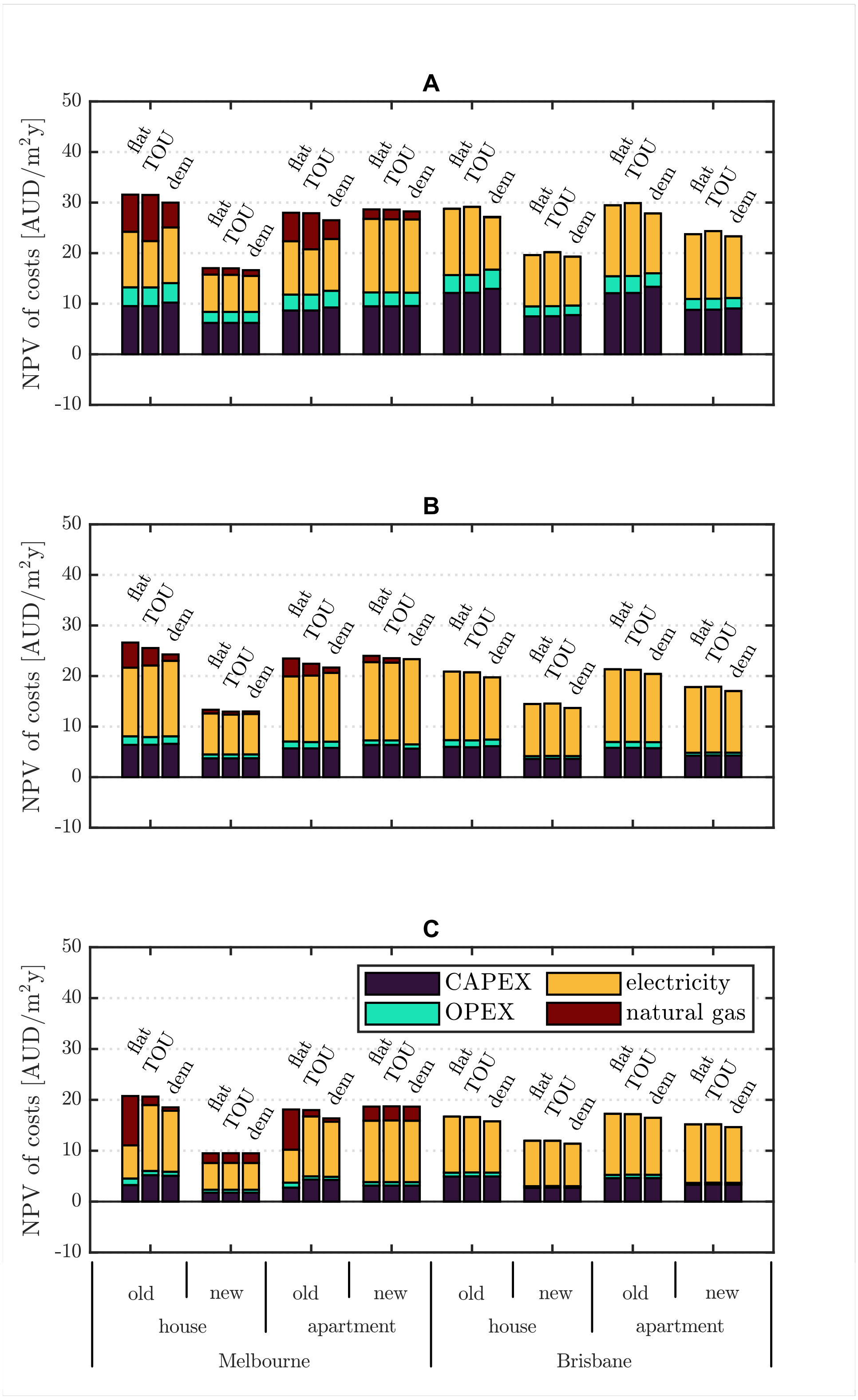

Figure S5: Annualised net present value (NPV) of total costs breakdown for cases without DER and various electricity tariffs, sites and building types, with tight setpoint without pre-heating/cooling (top), tight setpoint with pre-heating/cooling (middle), and loose setpoint with pre-heating/cooling (bottom).

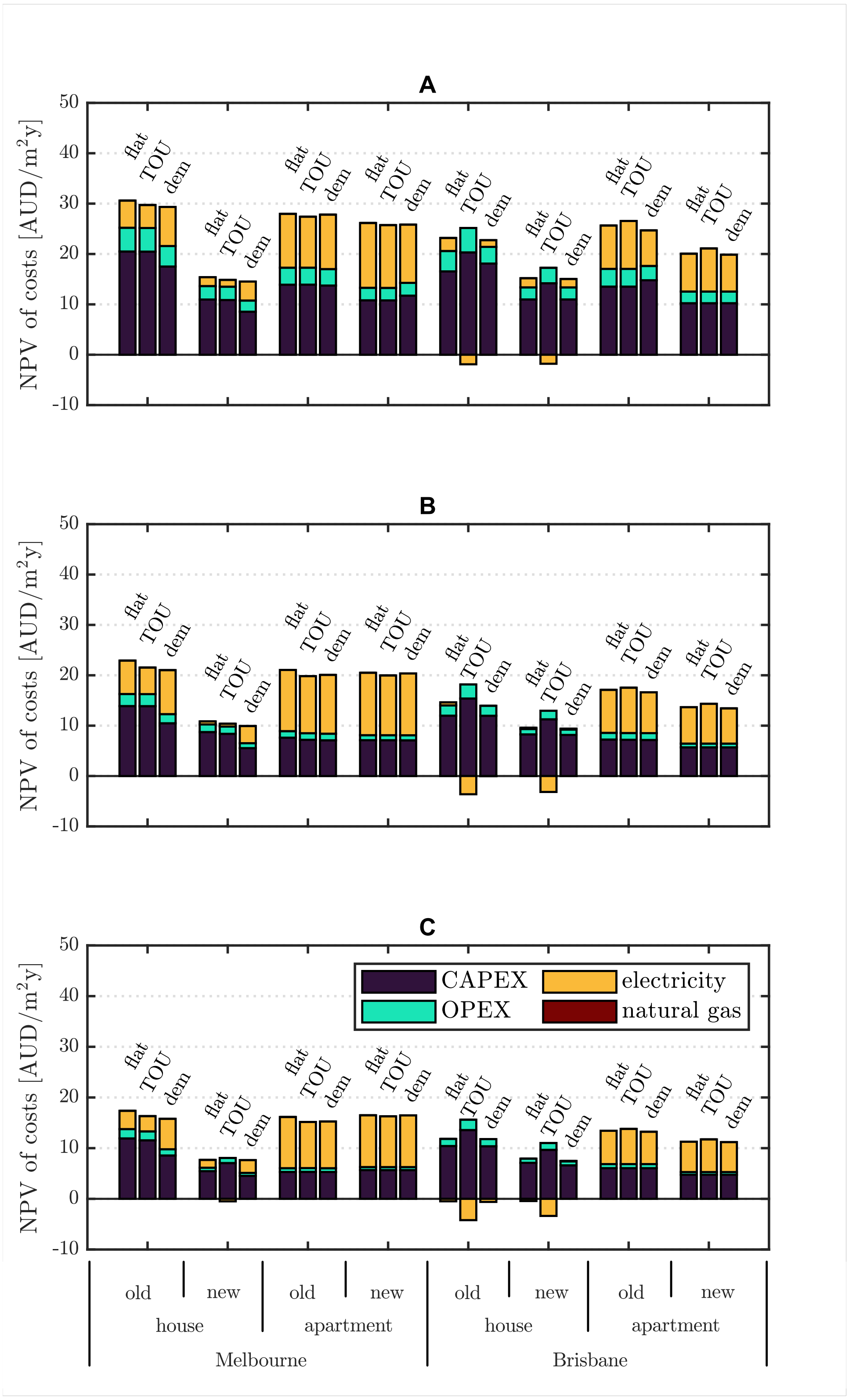

Figure S6: Annualised net present value (NPV) of total costs breakdown for cases with full electrification and DER and various electricity tariffs, sites and building types, with tight setpoint without pre-heating/cooling (top), tight setpoint with pre-heating/cooling (middle), and loose setpoint with pre-heating/cooling (bottom).

Table S1: Average cost savings from alternative TCM strategies relative to the case of a tight setpoint without pre-heating/cooling. Values were averaged over building vintage and electricity tariffs, for each site and building type.

TCM strategy	Case	Optimal*	Melbourne		Brisbane	
			House	Apartment	House	Apartment
Tight setpoint with pre-heating/cooling	No DER	D&O	20.2 %	25.6 %	29.0 %	25.6 %
Loose setpoint with pre-heating/cooling	No DER	D&O	39.7 %	37.5 %	42.8 %	37.5 %
Tight setpoint with pre-heating/cooling	Electrification and DER	D&O	28.7 %	28.0 %	33.5 %	28.0 %
Loose setpoint with pre-heating/cooling	Electrification and DER	D&O	46.8	39.1 %	45.4 %	39.1 %
Tight setpoint with pre-heating/cooling	No DER	O	1.7 %	3.3 %	3.9 %	3.3 %
Loose setpoint with pre-heating/cooling	No DER	O	10.4 %	9.1 %	10.8 %	9.1 %
Tight setpoint with pre-heating/cooling	Electrification and DER	O	5.4 %	3.3 %	2.6 %	3.3 %
Loose setpoint with pre-heating/cooling	Electrification and DER	O	12.9 %	8.6 %	8.3 %	8.6 %

* D&O: design and operation; O: operation only.

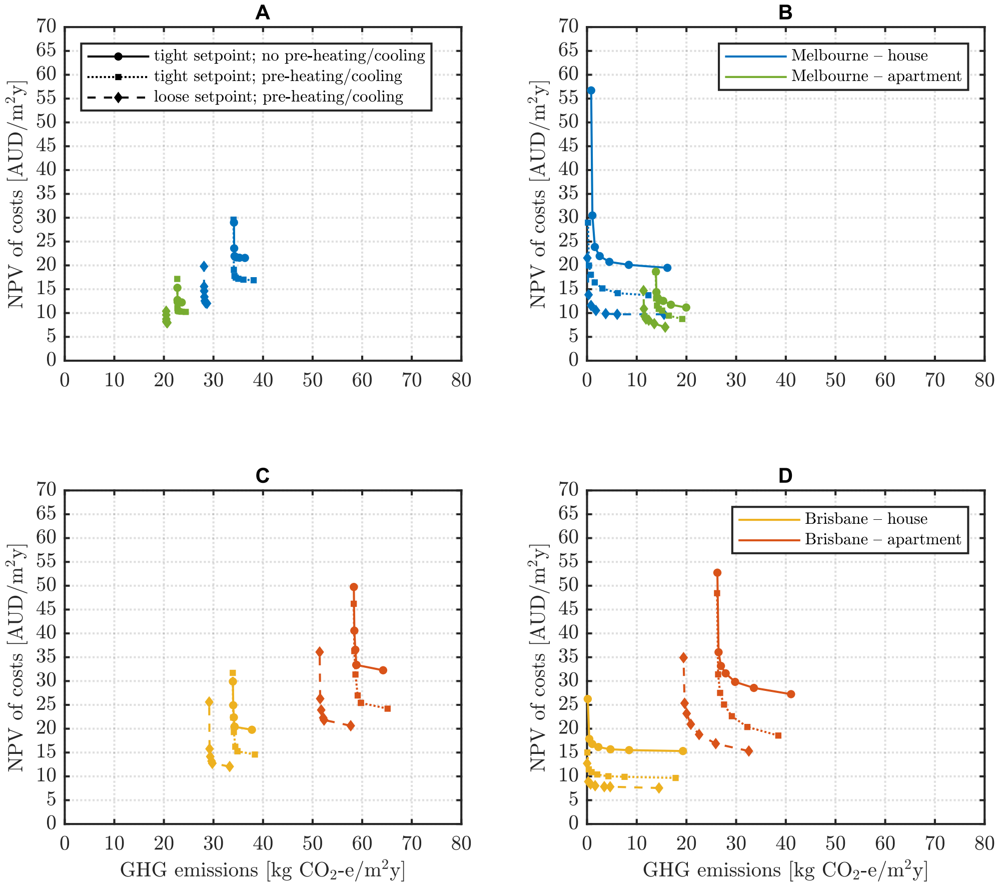

Figure S7: Trade-offs between annualised net present value (NPV) of total costs and yearly GHG emissions per unit building floor surface for cases without DER (left) and full electrification and DER (right) and a flat electricity tariff, for two new building types in Melbourne (top) and Brisbane (bottom) and alternative TCM strategies (solid, dotted and dashed lines).

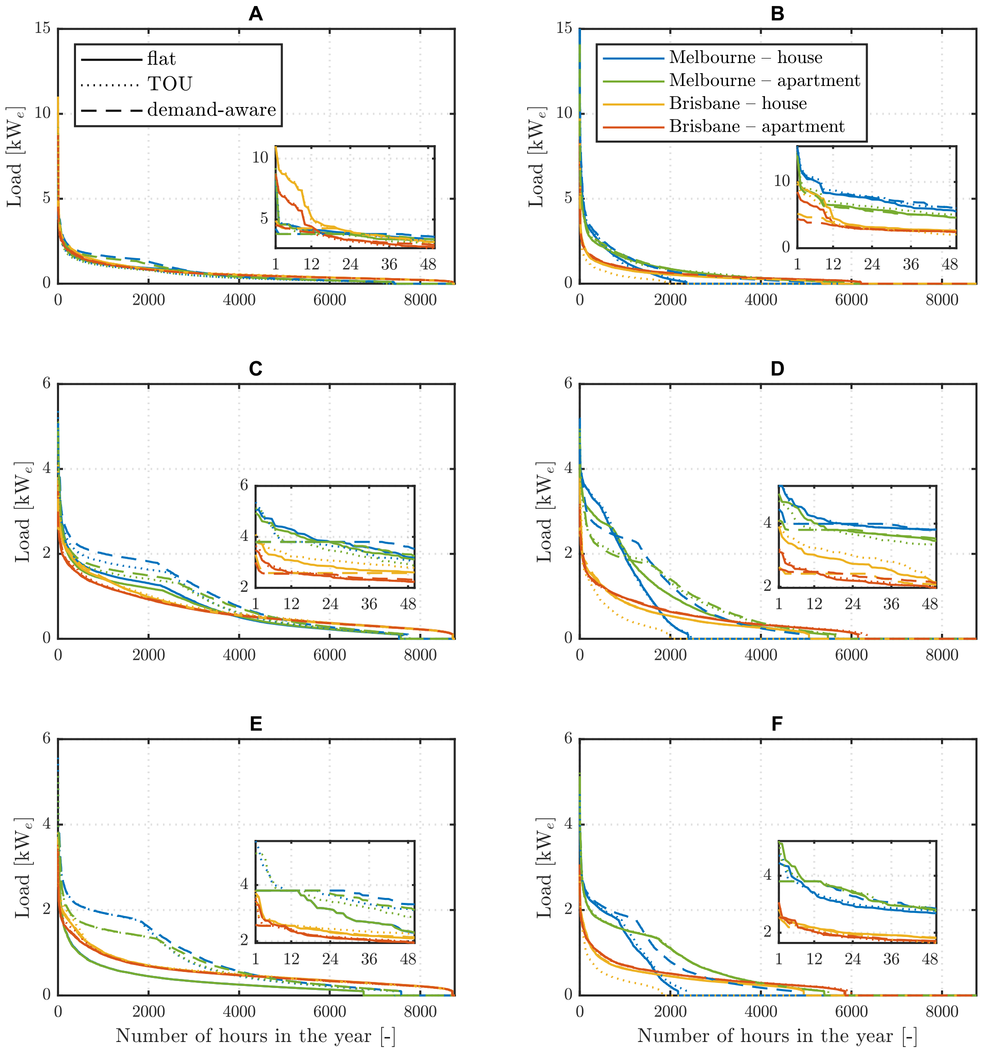

Figure S8: Yearly electricity load duration curves with detail of the highest peak loads for cases without DER (left) and full electrification and DER (right), four old building types, various sites and the three electricity tariffs with tight setpoint without pre-heating/cooling (top), tight setpoint with pre-heating/cooling (middle), and loose setpoint with pre-heating/cooling (bottom).

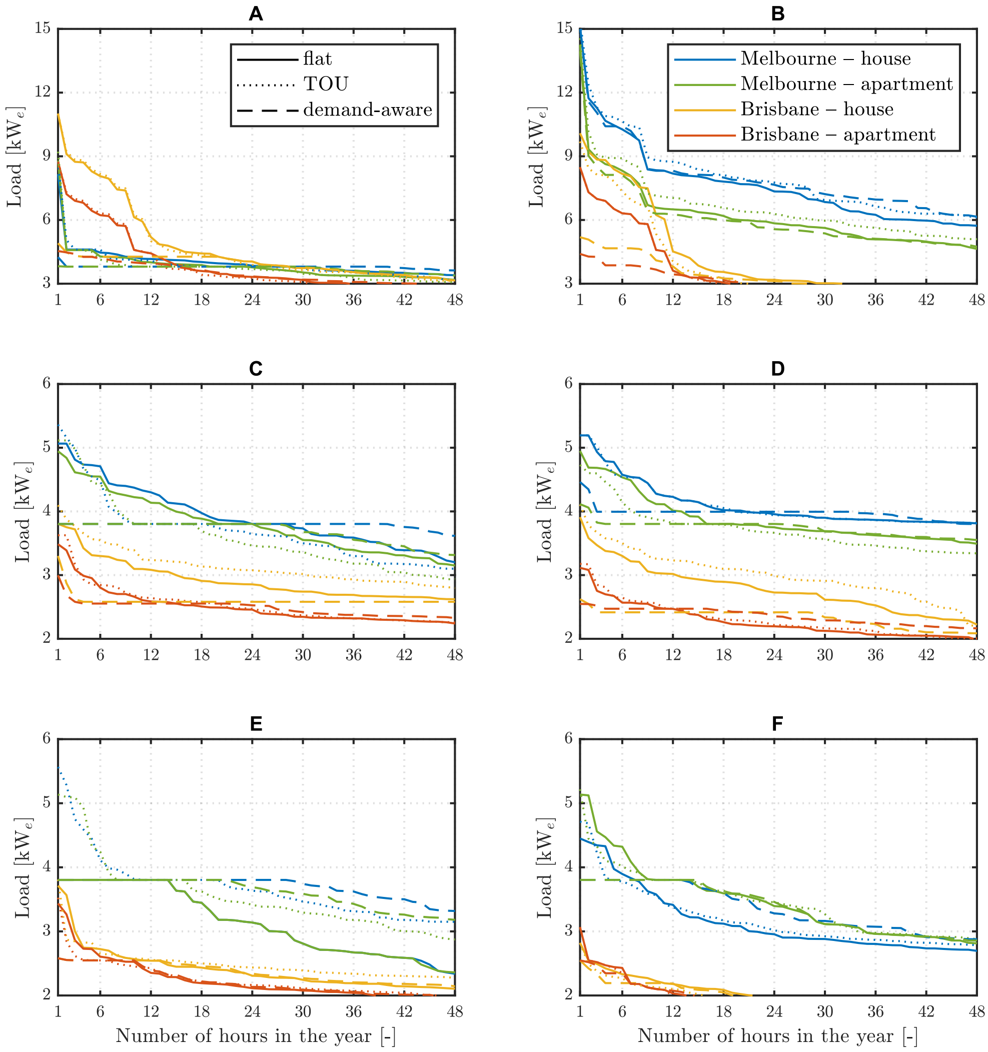

Figure S9: Electricity load duration curves of the highest peak loads for cases without DER (left) and full electrification and DER (right), four old building types, various sites and the three electricity tariffs with tight setpoint without pre-heating/cooling (top), tight setpoint with pre-heating/cooling (middle), and loose setpoint with pre-heating/cooling (bottom).

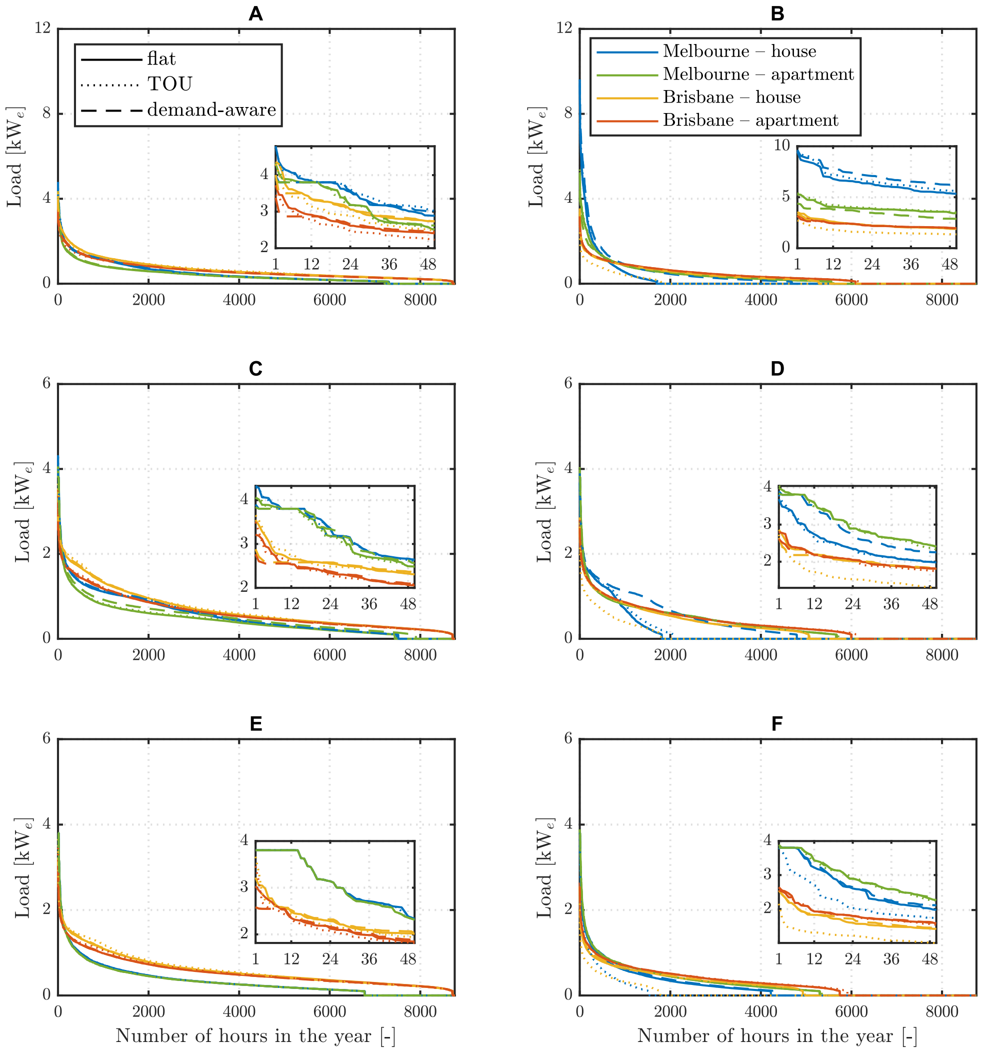

Figure S10: Yearly electricity load duration curves with detail of the highest peak loads for cases without DER (left) and full electrification and DER (right), four new building types, various sites and the three electricity tariffs with tight setpoint without pre-heating/cooling (top), tight setpoint with pre-heating/cooling (middle), and loose setpoint with pre-heating/cooling (bottom).

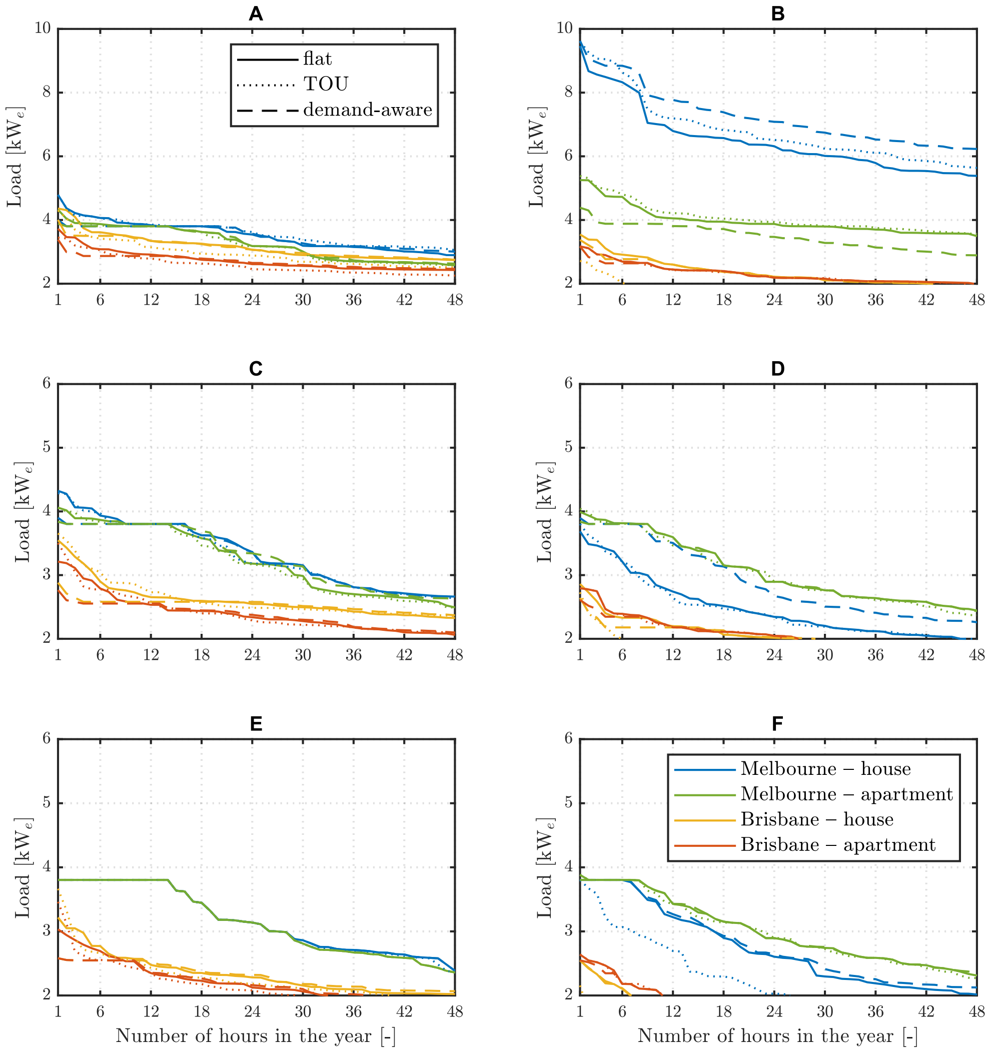

Figure S11: Electricity load duration curves of the highest peak loads for cases without DER (left) and full electrification and DER (right), four new building types, various sites and the three electricity tariffs with tight setpoint without pre-heating/cooling (top), tight setpoint with pre-heating/cooling (middle), and loose setpoint with pre-heating/cooling (bottom).

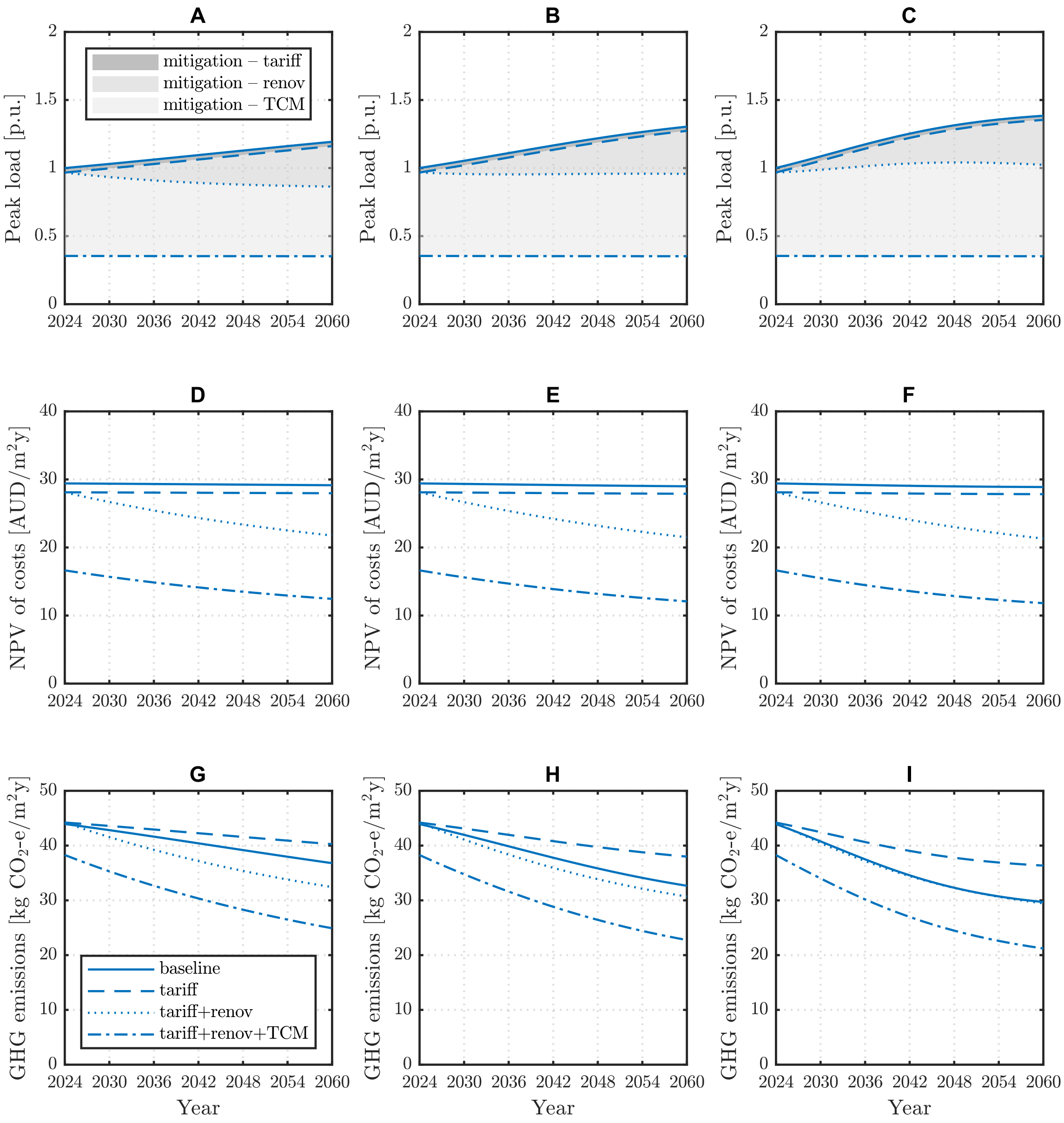

Figure S12: Aggregate peak load projections and mitigation potential of various strategies (top) average household annualised NPV of total costs (middle) and GHG emissions (bottom) for a low-density feeder in Melbourne for slow (left), medium (centre) and fast (right) paces of electrification and DER, and a 2.5 % per annum renovation of the building stock.

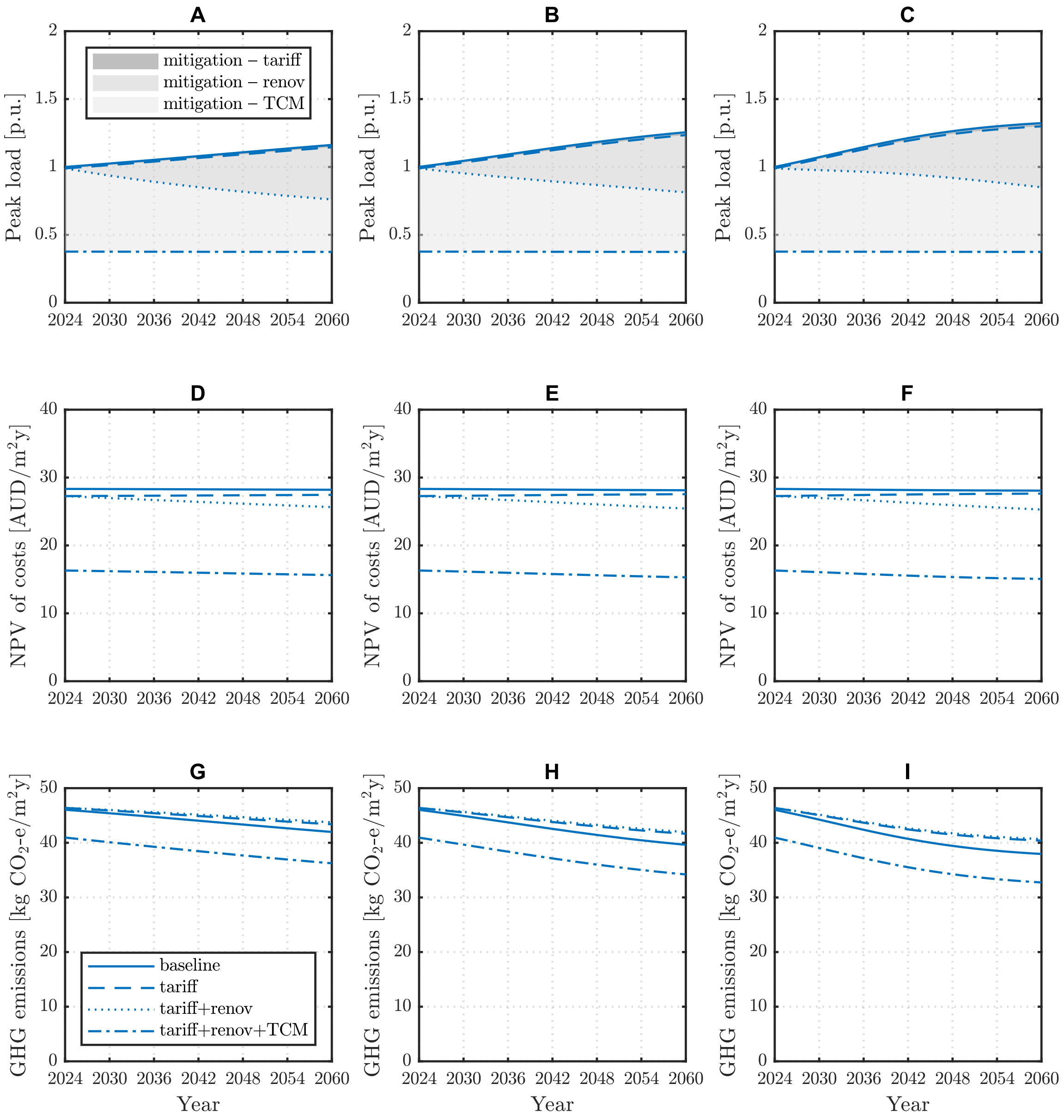

Figure S13: Aggregate peak load projections and mitigation potential of various strategies (top) average household annualised NPV of total costs (middle) and GHG emissions (bottom) for a high-density feeder in Melbourne for slow (left), medium (centre) and fast (right) paces of electrification and DER, and a 2.5 % per annum renovation of the building stock.

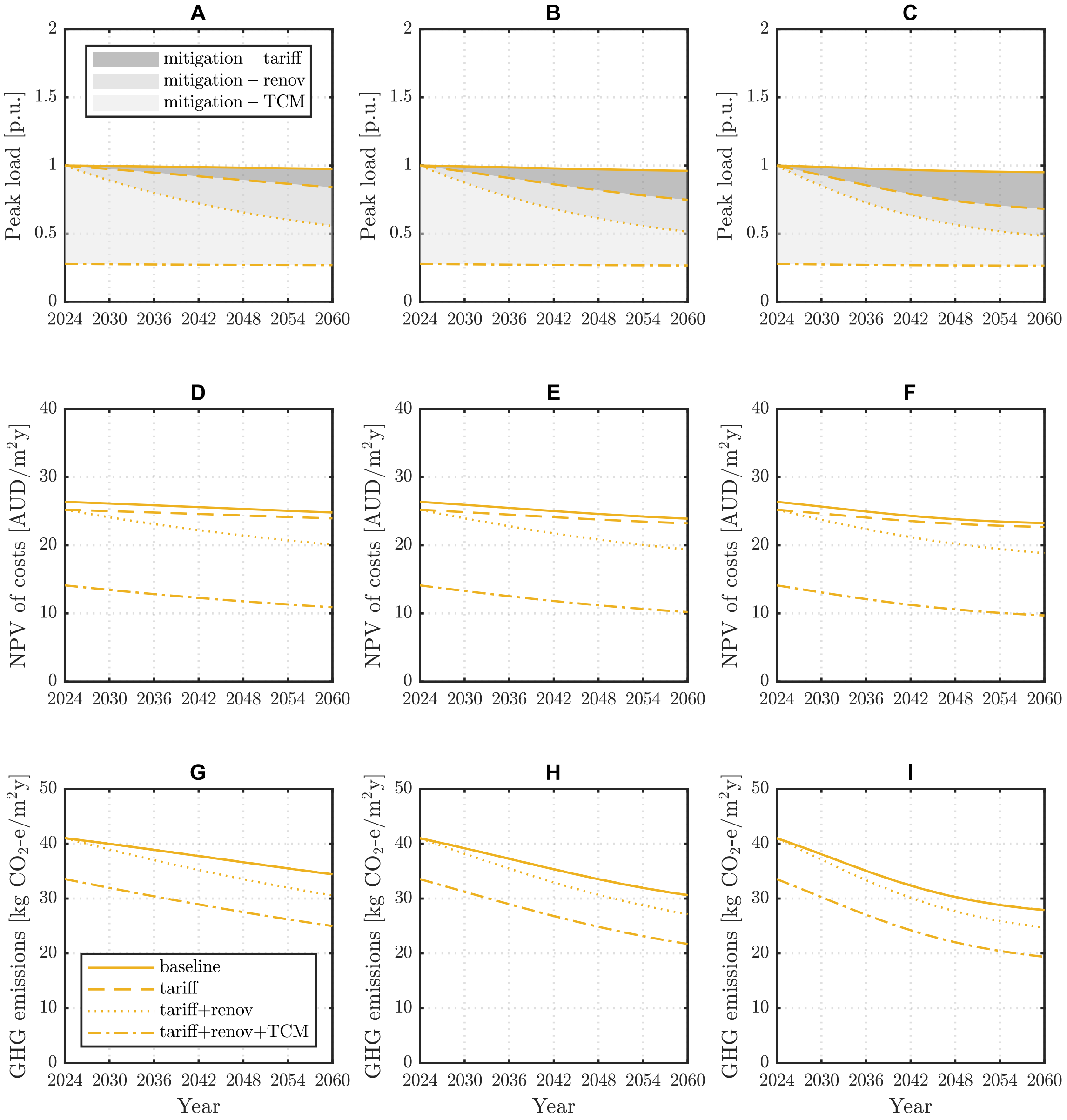

Figure S14: Annualised net present value (NPV) of total costs (top) and GHG emissions (bottom) projections for the average household supplied by a low-density feeder in Brisbane for slow (left), medium (centre) and fast (right) paces of electrification and DER and a 2.5 % per annum renovation of the building stock.

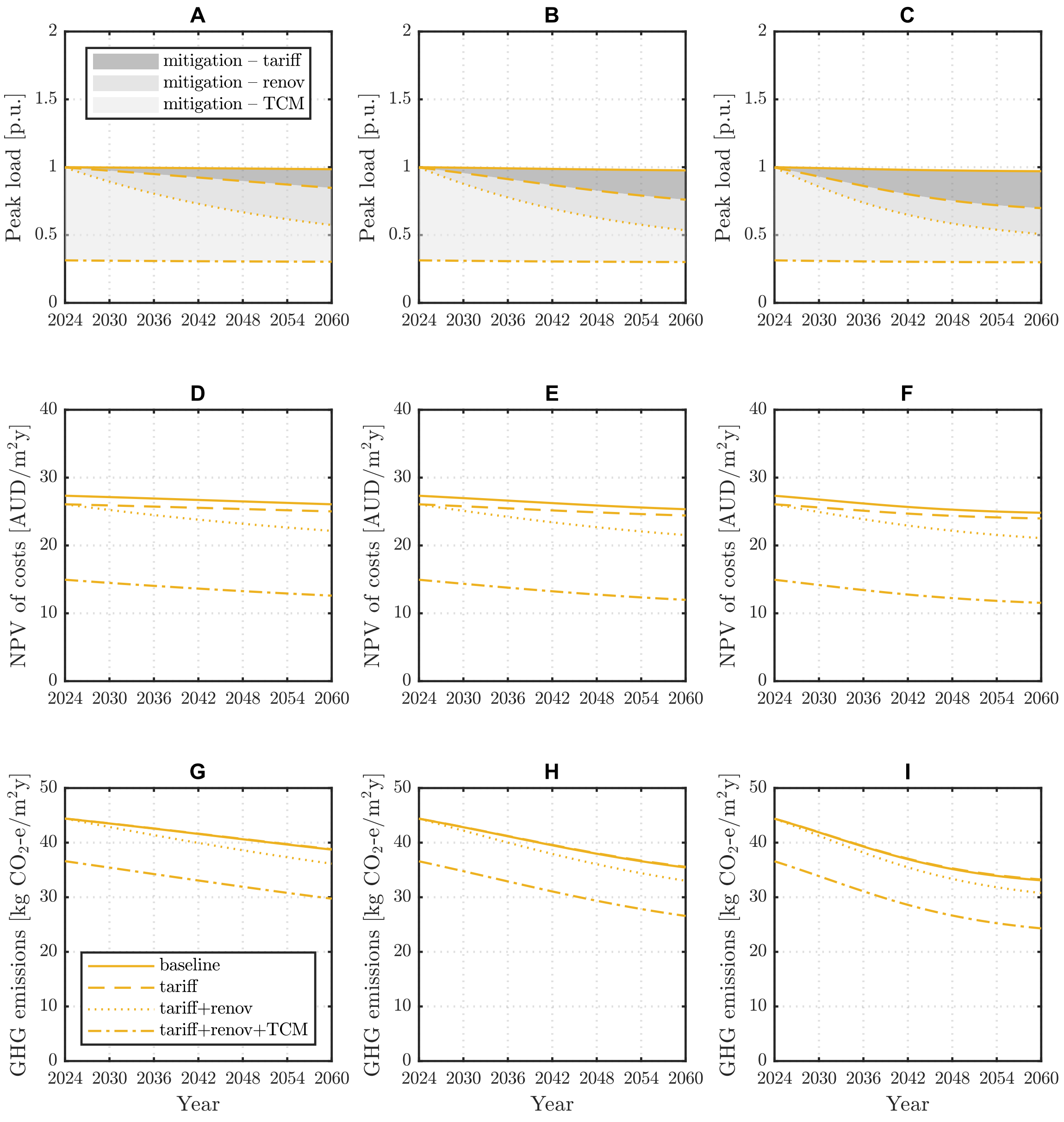

Figure S15: Annualised net present value (NPV) of total costs (top) and GHG emissions (bottom) projections for the average household supplied by a high-density feeder in Brisbane for slow (left), medium (centre) and fast (right) paces of electrification and DER and a 2.5 % per annum renovation of the building stock.

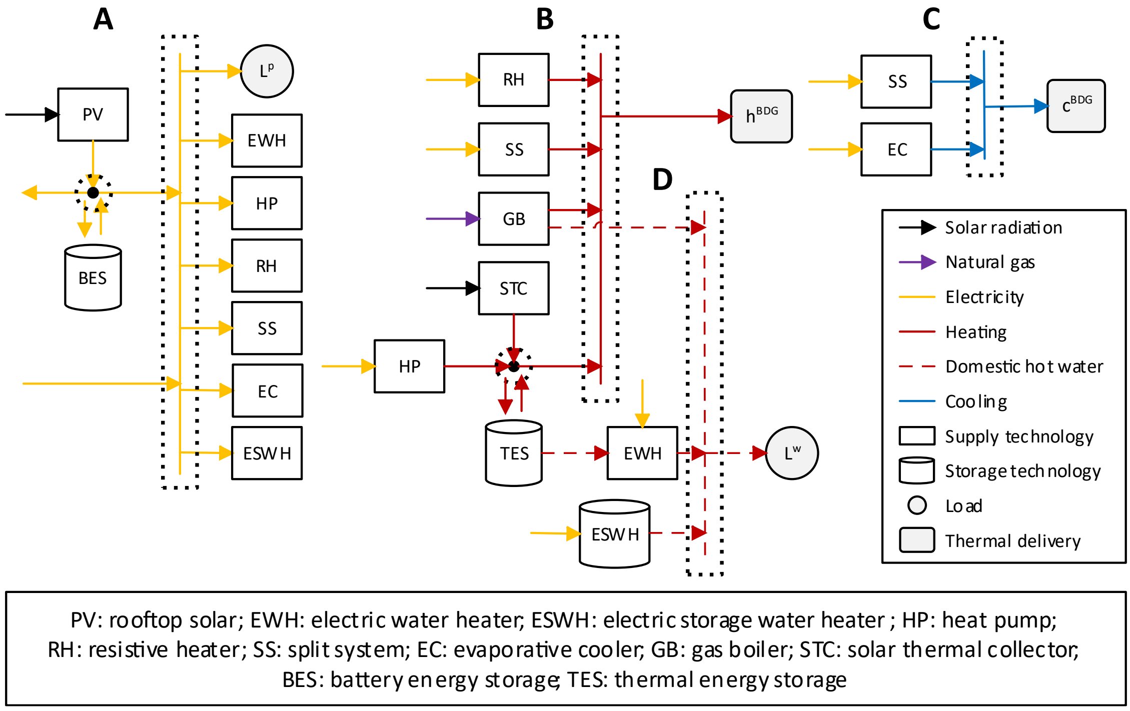

Figure S16: Household (A) electricity, (B) heating, (C) cooling and (D) domestic hot water supply systems and technologies considered in this work.

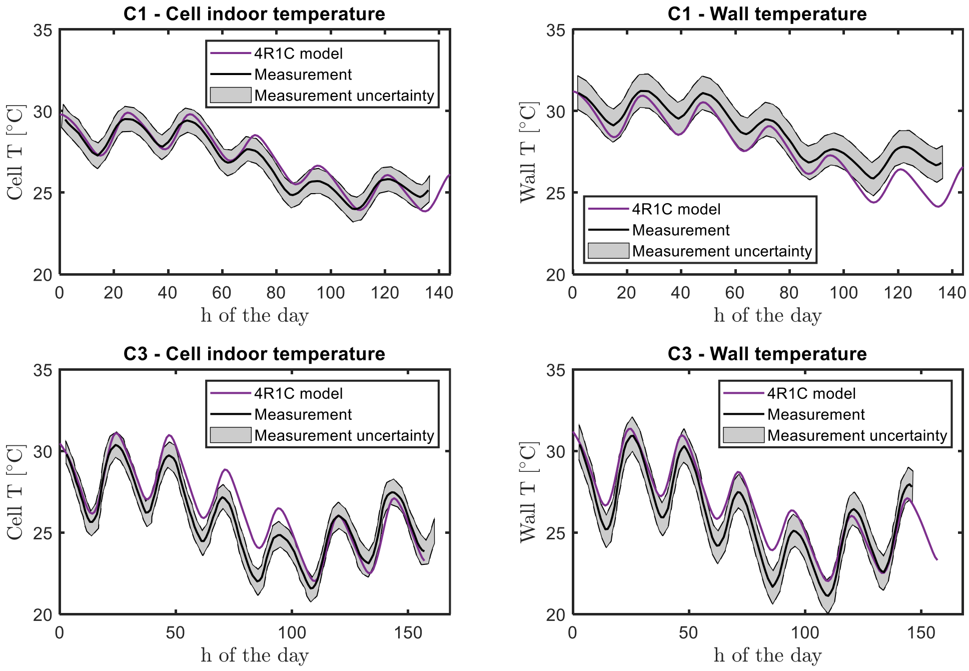

Figure S17: Dynamic building model validation: predicted indoor and wall temperature evolution by the 4R1C dynamic building model versus experimental measurements [1]

Table S2: Dynamic building model validation: error statistics for the developed 4R1C dynamic building model against experimental measurements [1]

Cell	Temperature reading	Maximum error [°C]	Minimum error [°C]	Average error [°C]	Error standard deviation [°C]
1	Cell	1.22	0.00	0.21	0.46
1	Wall	2.61	0.04	-0.92	0.55
3	Cell	2.39	0.00	0.60	0.90
3	Wall	2.25	0.05	0.76	0.77

Table S3: Value of charges on grid-purchased electricity from [2] or further elaboration of data therein, for the various electricity tariffs considered in this work.

Charge	Usage [c AUD/kWh]		Off-peak [c AUD/kWh]		Peak [c AUD/kWh]		Demand [c AUD/kW/y]	
**Site**	**Mel**	**Bri**	**Mel**	**Bri**	**Mel**	**Bri**	**Mel**	**Bri**
Flat tariff	27.3	21.7						
Time-of-use* tariff			21.5	16.3	38.6	33.1		
Demand-aware† tariff	16.4	13.0					25.9-136.8	29.1-153.3

* The average ratio of peak-to-off-peak charges reported by the Australian Energy Regulator, and a reference demand profile were used to compute these charges so that the yearly bill is equal to the case of a flat tariff at the state-averaged usage charge.

† The demand charge differs on a case-by-case basis to yield the same electricity bill as that of a flat tariff at the state-average usage charge; values of the demand charge remain within the reported range.

Table S4: Number of residential customers and their share in FY 2023-24 being subject to any of the electricity tariff structures considered in this work for the distribution network service providers active in Victoria and Queensland, Australia [3]

Distribution network service provider	State	Residential customers	Flat tariff	TOU tariff	Demand-aware tariff
AusNet	Victoria	728003	53%	47%	0%
CitiPower	Victoria	284461	77%	23%	0%
Jemena	Victoria	345429	79%	21%	0%
Powercor	Victoria	807541	65%	35%	0%
United Energy	Victoria	638904	82%	18%	0%
Energex	Queensland	1451545	46%	0%	54%
Ergon	Queensland	652100	51%	0%	49%
**Overall Victoria***		**2804338**	**69%**	**31%**	**0%**
**Overall Queensland***		**2103645**	**47%**	**0%**	**53%**

* Overall residential customers represent the total by state, whilst shares are averages weighted by the number of consumers served by each distribution network service provider active in that state.

Table S5: Shares of building types and vintage considered for feeders serving areas with different population densities.

Feeder	Building share [%]			
	Old house	New house	Old apartment	New apartment
Low-density	72	8	18	2
Medium-density	45	5	45	5
High-density	18	2	72	8

REFERENCES

1. Domínguez-Torres, C.A., Suárez, R., León-Rodríguez, A.L., and Domínguez-Delgado, A. (2022). Experimental validation of a dynamic numeric model to simulate the thermal behavior of a facade. Appl Therm Eng *204*. https://doi.org/10.1016/j.applthermaleng.2021.117686.
2. Australian Energy Regulator (2021). State of the energy market 2021.
3. Network pricing proposals and tariff variations | Australian Energy Regulator (AER) https://www.aer.gov.au/industry/networks/pricing-proposals-tariff-variations.
